# Conserving the Birds of Uganda’s Banana-Coffee Arc: Land Sparing and Land Sharing Compared

**DOI:** 10.1371/journal.pone.0054597

**Published:** 2013-02-04

**Authors:** Mark F. Hulme, Juliet A. Vickery, Rhys E. Green, Ben Phalan, Dan E. Chamberlain, Derek E. Pomeroy, Dianah Nalwanga, David Mushabe, Raymond Katebaka, Simon Bolwig, Philip W. Atkinson

**Affiliations:** 1 British Trust for Ornithology, Thetford, Norfolk, United Kingdom; 2 The Royal Society for the Protection of Birds, Sandy, Bedfordshire, United Kingdom; 3 Department of Zoology, University of Cambridge, Cambridge, United Kingdom; 4 Dipartimento di Biologiá Animale e dell’Uomo, University of Turin, Turin, Italy; 5 Department of Biological Sciences, Makerere University, Kampala, Uganda; 6 NatureUganda, Kampala, Uganda; 7 Department of Management Engineering, Technical University of Denmark, Copenhagen, Denmark; University College London, United Kingdom

## Abstract

Reconciling the aims of feeding an ever more demanding human population and conserving biodiversity is a difficult challenge. Here, we explore potential solutions by assessing whether land sparing (farming for high yield, potentially enabling the protection of non-farmland habitat), land sharing (lower yielding farming with more biodiversity within farmland) or a mixed strategy would result in better bird conservation outcomes for a specified level of agricultural production. We surveyed forest and farmland study areas in southern Uganda, measuring the population density of 256 bird species and agricultural yield: food energy and gross income. Parametric non-linear functions relating density to yield were fitted. Species were identified as “winners” (total population size always at least as great with agriculture present as without it) or “losers” (total population sometimes or always reduced with agriculture present) for a range of targets for total agricultural production. For each target we determined whether each species would be predicted to have a higher total population with land sparing, land sharing or with any intermediate level of sparing at an intermediate yield. We found that most species were expected to have their highest total populations with land sparing, particularly loser species and species with small global range sizes. Hence, more species would benefit from high-yield farming if used as part of a strategy to reduce forest loss than from low-yield farming and land sharing, as has been found in Ghana and India in a previous study. We caution against advocacy for high-yield farming alone as a means to deliver land sparing if it is done without strong protection for natural habitats, other ecosystem services and social welfare. Instead, we suggest that conservationists explore how conservation and agricultural policies can be better integrated to deliver land sparing by, for example, combining land-use planning and agronomic support for small farmers.

## Introduction

Increases in human population and per capita consumption are likely to lead to greatly increased agricultural demand over at least the next 40 years [1], which could lead to further habitat destruction, loss of ecosystem services, ecosystem simplification and species loss [2]. This has raised the question of how food production and biodiversity conservation can best be reconciled [3], [4], [5]. In temperate regions, and in some cases in the tropics, much emphasis has been placed on agri-environment and certification schemes to encourage wildlife-friendly farming, or land sharing, where lower-yield farming enables a high biodiversity to be maintained within farmed landscapes [6], [7], [8]. Land sharing has been championed by many in conservation practice and research (e.g. [9]–[11]) because wildlife-friendly farmland typically supports higher species richness and more species of the natural or semi-natural habitat it replaced than does intensively-managed farmland [12], [13]. However, some studies have cast doubt on the effectiveness of land sharing initiatives, in both temperate and tropical areas [14]–[16]. This is both because wildlife-friendly farmland often offers a poor substitute habitat, particularly for the most sensitive species, and because it often entails a yield penalty and thus requires a greater area to produce any given amount of food (but see criticism of this interpretation of the evidence in [17]).

An alternative proposal to land sharing is land sparing, where agricultural land is farmed to produce a high yield of crops. This requires a smaller area of land than would be needed to grow the same total production target by lower-yielding methods. If this spared land is maintained or restored as natural habitat, then species associated with natural habitats are expected to benefit [6]. Future realised agricultural yield is likely to have a strong effect on the amount of land demanded by a growing and increasingly affluent population in the developing world [18], [19] so the land sparing approach appears to be a strategy worth considering. There is a range of possible intermediate strategies but, for typical species assemblages, attempting to combine land sparing and land sharing benefits fewer species than adopting the better of the two pure strategies [4], [6]. The pertinent question for policy-makers and conservationists is the extent to which conservation resources should be allocated towards preventing habitat loss, relative to ameliorating the negative impacts of intensification.

Explicit comparisons between expected biodiversity outcomes under land sparing and land sharing approaches are rare and more are needed from a wider range of locations in order to inform the debate on this issue [20]. A study on the responses of bird and tree species to varying agricultural yields in forested regions in Ghana and India found that many species were expected to have larger total population sizes with high yield farming combined with land sparing so that more forest was retained, than with low yield farming and land sharing [4]. This was particularly the case for species expected to have smaller total populations with, than without farming present (losers). In this paper, we perform a similar analysis of data on population densities of birds as a function of yield in the form of income and food energy collected across a large region in southern Uganda which includes both forest and agriculture. We use comparable methods to those used in Ghana and India [4] to assess whether the conclusions from those studies also apply to birds in Uganda.

## Materials and Methods

### Ethics Statement

This research was conducted through the NGO NatureUganda which has a MoU allowing research to be carried out in the majority of forest sites which were managed by the National Forestry Authority and the Uganda Wildlife Authority. Permission to access privately owned land (all farmland sites and one forest site) was given by the land owners. Some forest sites were part of the Mabira Forest Reserve, managed by the National Forestry Authority.

### Study Area

The study area lies within the banana-coffee farming system in the Lake Victoria crescent, southern Uganda, a farming landscape covering more than 50,000 km^2^. The area is one of high human population density and good access to infrastructure and markets. There are two wet seasons per year and annual rainfall is between 1000–1500 mm, making it one of the wetter regions of the country [21], [22]. Major land uses include perennial crops, mainly banana and coffee, but there is an increasing shift towards cultivation of annual crops, largely in response to emerging disease and pest issues associated with traditional coffee and banana production. Land under agriculture increased by 11.4% between 1975 and 2000 in the wider region [23] and deforestation trends have been high across Africa in recent years [24]. Whilst it is likely that some forest fragments in Uganda have been isolated within savanna for hundreds or thousands of years other patches will have been part of much larger areas of forest before extensive forest clearance, particularly in the twentieth century, for timber, agriculture and as a measure against sleeping sickness [25].

**Figure 1:**
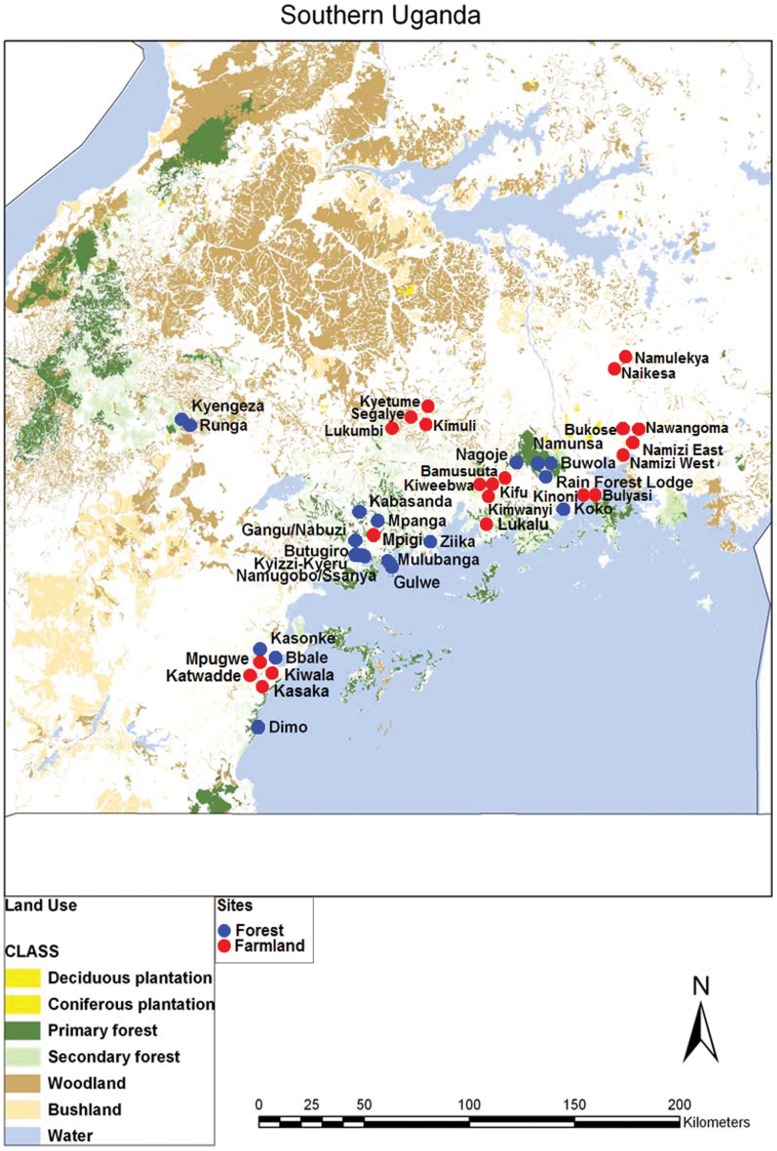
Location of study sites in Uganda. Forest sites are denoted with blue circles, farmland sites with red circles.

The farmland study was conducted at 22 sites, each consisting of a 1 km x 1 km square, selected to represent a broad range of agricultural land uses from small-scale mixed holders to large-scale monoculture plantations. Population density for southern Ugands was derived from the 2002 Uganda National Census (URL: www.ubos.org) and was used as a surrogate for cultivation intensity with sites selected across a population gradient. Forest sites were selected from native forest patches within the farmed landscape described above. Forest sites were limited by the availability of patches of sufficient size and thirty forest patches of at least 1 km^2^ in area were identified from the Biomass Map of Uganda [26]. Each of these sites was visited in November 2007 in order to determine (i) whether the forest patch still existed (ii) the extent of degradation and (iii) whether there were any access problems. Ten forest sites that had large clear-felled areas for cultivation or charcoal burning, and that therefore had open canopies (all sites <50% canopy cover), were excluded from the study. Of the remaining 20 forest sites selected for the bird surveys, one was partially deforested between the first visit and the commencement of bird surveys and was therefore also excluded, leaving 19 sites (Table 1). A map of site locations is given in Figure 1.

**Table 1:** Sites surveyed, area of forest sites, effort (number of point counts conducted) and annual yield per hectare (GJ ha^−1^ year^−1^ and US$ ha^−1^ half year^−1^).

Site	Habitat	Area of forest(ha)	Forest perimeter(km)	Effort (Point countsconducted)	Food Energy(GJ ha^−1^ year^−1^)	Income(US$ ha^−1^ year^−1^)
Bamusuuta	Farmland			50	8.736	431.26
Bukose	Farmland			50	13.172	555.26
Bulyasi	Farmland			50	5.43	510.92
Kasaala	Farmland			50	10.296	526.68
Katwadde	Farmland			50	7.342	414.98
Kifu	Farmland			40	11.13	312.9
Kimuli	Farmland			50	3.614	374.42
Kimwanyi	Farmland			50	8.958	370.02
Kinoni	Farmland			50	4.416	448.48
Kiwaala	Farmland			50	6.488	338.3
Kiweebwa	Farmland			50	8.346	330
Kyetume	Farmland			50	2.554	340.2
Lukalu	Farmland			50	4.784	483.14
Lukumbi	Farmland			50	8.696	546.84
Mpigi	Farmland			50	6.276	591.8
Mpugwe	Farmland			50	12.124	477.24
Naikesa	Farmland			50	17.516	583.1
Namizi East	Farmland			50	10.256	556.34
Namizi West	Farmland			50	14.128	589.98
Namulekya	Farmland			50	22.84	579.48
Nawangoma	Farmland			50	24	667.68
Segalye	Farmland			50	2.63	407.74
Bbale	Forest	5.27	40.21	16	0	0
Butugiro	Forest	10.22	89.85	20	0	0
Buwola	Forest	70.78	26.96	20	0	0
Dimo	Forest	17.19	93.23	20	0	0
Gangu/Nabuzi	Forest	19.77	97.32	14	0	0
Gulwe	Forest	21.74	71.01	6	0	0
Kabasanda	Forest	24.62	128.15	20	0	0
Kasonke	Forest	1.91	13.32	12	0	0
Koko	Forest	14.7	114.1	12	0	0
Kyengeza	Forest	27.05	129.73	8	0	0
Kyizzi-Kyeru	Forest	10.27	82.01	16	0	0
Mpanga	Forest	17.69	104.99	20	0	0
Mulubanga	Forest	27.26	90.39	7	0	0
Nagoje	Forest	35.83	75.07	20	0	0
Namugobo/Ssanya	Forest	11.6	78.79	20	0	0
Namunsa	Forest	67.77	55.53	20	0	0
Rain Forest Lodge	Forest	36.78	77.27	20	0	0
Runga	Forest	5.71	28.01	6	0	0
Ziika	Forest	9.99	111.7	8	0	0

### Bird Surveys

At each site a folded line transect of 2 km in length was followed, beginning at a random location and following paths and tracks where this was necessary to avoid trampling crops and for ease of access. Point counts [27] were located at 200 m intervals along each transect, totalling 10 per site for farmland but often fewer for forest, depending on the size of the forest patch. After a preliminary visit to the forest sites, low bird activity was apparent within a short time of arrival of the observer at the survey point. This is thought to have been caused by the noise generated by moving towards the point through forest vegetation and the lack of habituation to people compared to farmland birds; this effect did not appear to occur in the more open habitat on farmland. As a result, a 2-minute settling period was used before the 10-minute bird recording period began in forest, but no settling period was considered necessary in farmland. A comparison between forest points with and without a settling period indicated that this difference in methods might have made a difference to two species observed in both forest and farmland, but that the relationship was weak and would have made, at most, a very small difference to the results of the analysis presented here (Text S1, Table S1). Birds were recorded during the 10-minute survey period and each record assigned to one of three distance bands (<25 m, 25–50 m, >50 m) according to the distance from the point to the location of the bird when it was first detected. Distances were estimated by eye, but with regular checks against directly measured distances. Birds first seen when in flight were recorded separately. Farmland points were visited five times between February 2006 and January 2007, with intervals of at least six weeks between visits. Forest points were visited twice between February and April 2008.

### Habitat Surveys

Five 1 km parallel transects were arranged from east to west across each farmland site, separated by 200 m. Between February and June 2006 the length of each transect passing through different vegetation and crop types was measured using a tape measure. Vegetation types recorded were: cultivated, fallow, woodlot, homestead (building and yard where people and domestic animals reside), road, managed pasture, unmanaged pasture, school/market place, kraal, garden, natural vegetation and whether or not this was forest. The list of crops used for yield calculation is given in Table 2. The proportion of total land covered by each crop was estimated as its proportion of the total length of transect.

**Table 2:** Energy of edible mass and % inedible refuse (skins, husks, stalks *etc*.) for crops for which yield and area data were available.

Crop	Refuse (%)	GJ tonne-1
Banana	36	3.71
Bean	0	13.93
Cabbage	20	1.03
Cassava	14	6.67
Coffee	0	0
Eggplant	19	1.01
Groundnut	0	23.74
Maize	0	15.27
Millet	0	15.82
Pineapple	49	2.09
Pumpkin	30	1.09
Rice	0	14.98
Simsim	0	23.97
Sorghum	0	14.18
Soy bean	0	18.66
Spinach	28	0.97
Sugarcane	52	2.11
Sweet potato	28	3.59
Tea	0	0
Tomato	9	0.75
Vanilla	0	0
Yam	14	4.94

### Estimation of Crop Yield for Farm Income and Food Energy Measures

To evaluate the potential performance of land sparing and land sharing we need to model the total population size of species whilst achieving a given fixed level of agricultural production (the production target) at a range of yields of food energy or farm income per unit area of the farmed landscape. This section describes how we estimated food energy and income yield from surveys.

### Biomass Yield of Agricultural Products

We estimated the biomass yield of crops in each of our study areas as an intermediate step in obtaining yields in terms of food energy and farm income. The conversion of biomass yield to food energy and income yield is described in later sections. Direct measurements of biomass yield were impractical because of the large number of study areas and their large size. Instead, reported yields from farmer interviews were used. Ten farmers at each site were interviewed in 2007 and 2008 about their cropping practices. For each site a list was drawn up, in conjunction with a local leader, of about 20 households. Those farmers with less than 50% of their land in production were removed from the list. The remaining famers were categorised according to the three most common crop types grown in the square kilometre. Up to 10 farmers were selected to ensure each of the major crop types in the area were included with farms broadly typical of the area. Six individual farmers/households were selected at random from the list of 10 farmers for interviewing. Within the selected households, the head, spouse or any other knowledgeable person was the target respondent. The crop yield information they reported was collected separately for two 6-month growing periods: April - August and September - March. The local units given were converted to tonnes. Biomass yield per unit area was calculated for each farmer, crop and growing season in tonnes ha^−1^. A number of very high yields were reported. For example, the highest recorded maize yield in Season 2 of 62 tonnes ha^−1^ was 15.5 times the next highest recorded yield for that season of 4 tonnes ha^−1^, so we assumed that the higher value was inaccurately reported. For crops where outliers of this kind were present (banana, cassava, maize, tomato and sweet potato) values above the 95^th^ percentile were excluded for both seasons. The yield per hectare for each crop at a site was taken to be the sum of the average yields for the two growing seasons. Pasture was a very small proportion of overall land use (1.41% of land under managed pasture, 2.41% under unmanaged pasture) and livestock productivity was not included in agricultural yield calculations.

### Farm Income

For estimating the yield for food and non-food products monetary currencies are an appropriate measure. The potential income generated per unit area of the farmed sites was calculated, by multiplying the biomass yield per hectare per year, calculated as described above, by the local market price per unit weight of each crop, which was obtained as a mean from market surveys in both seasons and for all clusters with farmed sites. The mean farm income for each crop at each site was then calculated by multiplying the mean value of a crop per hectare by the area of that crop at the site, separately for each of the two seasons. Incomes were then summed across all crops and divided by the total area of the site to give total income per unit area per season, which was converted to US$ ha^−1^ at 2007 exchange rates of 1 US$ = 1690 Ugandan Shillings (URL: WWW.OANDA.com: accessed on 01/11/2011). The values for the two seasons were then summed. Mean farm incomes were therefore estimates of the potential income per hectare of the whole farmed landscape per year which might have been derived from the crops grown, regardless of whether they were sold, bartered or consumed by the farmers, their families or livestock. Farm income for forest sites was assumed to be zero.

### Food Energy Yield

Food energy, unlike income, is not affected by market fluctuations. However, it is not as appropriate for products which have a high monetary value but low food energy, such as coffee and vanilla. Hence, we use both food energy and income yield in our analyses to check whether conclusions about the responses of bird densities to yield are robust to the choice of measures, neither of which is perfect. The amount of food energy contained in each crop per unit biomass harvested was assessed using values obtained from the literature for the energy content per unit weight of processed crop and the average proportion by weight of the harvested crop which is discarded as inedible refuse during the preparation of the crop for consumption, such as skin and husks. The values obtained for the crops present on transects are shown in Table 2. Values for most raw crops which occurred on the farmland transects were obtained from the United States Department of Agriculture (USDA) National Nutrient Database for Standard Reference (URL: http://www.nal.usda.gov/fnic/foodcomp/search/: accessed on 01/11/2011). The details of the data and methods used to calculate the energy content of various raw and processed foodstuffs are found in the project documentation [28]. For coffee the energy value derived from black coffee was assumed to be negligible so coffee was not considered to contribute to food energy yield. Similarly vanilla, a flavouring which is used in very small quantities, contributes negligibly to energy intake and was not considered in energy yield calculations. For sugar cane the proportion of refuse was determined from the percentage of fibrous bagasse and liquid juice and percentage of sugar in the juice [29], and the energy content calculated using USDA data for raw sugar. The energy value of edible food per unit of harvested biomass was then multiplied by the biomass yield per hectare minus the refuse and the area of each crop at each site to give total food energy production per site per season. Energy production values were then summed across all crops and divided by the total area of the site to give total food energy yield per unit area per season, expressed as GJ ha^−1^. The values for the two seasons were then added together.Food energy yields were therefore estimates of the food energy which might have been derived from the crops grown per hectare of the whole farmed landscape per year, regardless of whether they were sold, bartered or consumed by the farmers, their families or livestock. Food energy yields for forest sites were assumed to be zero.

### Data Analysis

#### Species densities

Records of birds in flight and over 50 m from the point were discarded. Some aerial species, such as swallows and martins, were only seen in flight and are therefore not included. Eligible records were pooled across survey points for each site and each species and the number of point counts at each site was recorded and is shown in Table 1. Twenty point counts were performed at most forest sites (10 points visited twice) and 50 counts for most farmland sites (10 points visited five times). Effort and area for each site are shown in Table 1. Distance version 6.0 [30] was used to estimate detection probabilities for each species. Since there were only two distance bands, limiting the number of key parameters available for use to one, the half normal function with no adjustment terms was used for all species. For each species, point habitat type (forest or farmland), was included as a covariate if at least 20 individuals had been recorded in both habitats and this model was chosen if the AIC value was lower than that for the model with no covariate. For three species the habitat model had the lowest AIC but failed to converge so the model that ignored habitat type was used. For another species the habitat model had the lowest AIC but the variance estimation was invalid so the non-habitat model was used. Due to the low number of residual degrees of freedom goodness of fit tests were not possible so models were accepted based on visual determination of the plausibility of detection functions and the variance of the overall density estimate. Density values were estimated for each site with a detection function by habitat where habitat was selected as a covariate [31].

Too few registrations were available for some species to estimate a detection function from only the data for that species. These species were each assigned a detectability group depending on our assessments of their diet, habitat stratum and activity level. Species were classified as carnivore, frugivore, granivore, insectivore or omnivore and further classified as to which stratum of vegetation they usually inhabit by allocating each to one of five classes: 1) canopy or in sub-canopy of forest, or in canopy of large trees in other habitats, perch high in the canopy, 2) lower or middle layers of vegetation in forest or other habitats with dense tree cover 3) bushes or small trees, usually in open habitats 4) on the ground in open areas 5) low vegetation, often heard rather than seen. Species’ activity was classified as usually active (e.g., sunbirds) or usually static (e.g., kingfishers). Some groups were further aggregated to achieve an adequate sample size. Using these classifications 26 groups were formed which included all species recorded and which had sufficient observations to calculate detection functions. Density values were estimated by stratifying by species and estimating density by site. Habitat was included in some of these group models as a covariate using the same model selection process as for individual species above.

#### Fitting of density-yield curves

Parametric models were fitted to relate bird density to yield, as described previously (see supplementary online material in [4]). The method is summarised here. Univariate parametric Poisson regression models were fitted for each species by a maximum-likelihood (M-L) method. The dependent variable was population density, determined using Distance, with each of the two measures of yield being the independent variable. The following two alternative model formulations were used:

Model A
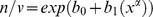



Model B
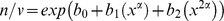
where *n* is the number of individuals of that species recorded, *v* = (*a* × *e*), where *a* is the effective detection area per survey point from Distance and *e* is effort (number of point counts conducted at the site). Note that *a* differed between farmland and forest sites for those species for which a habitat-specific detection function was used in Distance. The variable *x* represents yield (in either GJ ha^−1^ year^−1^ or income ha^−1^ year^−1^), and *b_0_*, *b_1_*, *b_2_* and *α* are constants estimated from the data. The value of *α* was constrained to be positive and not to exceed 4.6. This maximum value of *α* was used because for species with high *α*, the likelihood of the data was usually approximately constant with increasing *α* beyond this value, making a precise M-L model impossible to identify. However, the shape of the functions determined by the models with high *α* varied little as *α* was changed. These model formulations were selected because they give a wide range of shapes of curves. In particular, the M-L Model B curves were often hump-shaped, but with an asymmetrical shape. This asymmetry was visible in plots of density against yield for many species and was well described by the inclusion of the shape parameter *α*. For each square, the expected density under either Model A or Model B was calculated for a given set of parameter values and multiplied by the value of *v* for that square to give the expected number of individuals for that square. The natural logarithm of the Poisson probability of the observed number of individuals for the square, given the expected number under the model, was then obtained and summed across all squares to give the log-likelihood of the data. This log-likehood was then maximised to give M-L values of *b_0_*, *b_1_*, and *α*, for Model A and *b_0_*, *b_1_*, *b_2_* and α for Model B. Under Model B, the best-fitting hump-shaped functions sometimes had a high peak density value in a gap between groups of sites in the distribution of the yield variable. For some species, this peak density was much larger than the observed density at any site: sometimes thousands of times larger. We considered such models to be unrealistic and therefore constrained the model parameter values to give peak densities no greater than 1.5 times the maximum observed density. The maximised log-likelihoods were multiplied by −2 to give the residual deviances for models A and B. If the residual deviance for Model B was more than 3.84 (*Χ* ^2^ with 1 degree of freedom for *P = *0.05) lower than that for Model A then Model B was selected. Otherwise Model A was selected for reasons of parsimony. For species which were only observed in forest sites no model was fitted and a simple step function was assumed with the only non-zero density value density at zero yield in forest.

Densities for all species were also estimated for forest and farmland by Σn/Σv, with summation across all sites in each habitat and n and v as defined above.

#### Model of population size of a species in relation to yield and production target

We used a model developed previously [6], and used in Ghana and India [4], in which the expected total population of a species within a region is given by adding its total population in forest to its total population on farmed land. The expected population in forest was calculated as the product of the area of forest and the density of the species in forest obtained from its density-yield function. For a scenario in which the whole site was covered by forest, the total population is given by the density in forest, taken from the fitted density-yield curve, multiplied by the total area of the region. This is referred to as the baseline population and all other calculated total population sizes are expressed as proportions of the baseline population. The total population of the species on farmed land was calculated as the product of the area of farmed land in the region under a given farming scenario and the population density derived from the fitted density-yield function, given the yield assumed in that scenario. The impact of land allocation strategies on species populations was assessed at a defined level of production of food energy or income, referred to as the production target [6]. The production target can be produced at any yield per unit area of farmed land within a range defined by minimum and maximum permissible yields. The minimum permissible yield is that obtained by dividing the production target by the total cultivable area of the site. At lower yields than this, the amount of energy or income produced would be less than the production target. A maximum permissible yield is assumed set by maximum feasible levels of crop production, here designated as 1.25 times the maximum yield observed for the set of farmland sites we studied. This multiplier is arbitrary, but it has been shown previously that conclusions are robust to variation in the multiplier [4]. Within the permissible range of yields, the area of farmed land is obtained by dividing the production target by the assumed yield. The area of forest is assumed to be the remaining area of the region that is not required for farming and can therefore be obtained by subtracting the area of farmed land needed to grow the production target from the total area of the region. Hence, for a given production target, areas of farmed land and forest and the population density of the species on farmed land can be calculated for all yields within the permissible range. The total population of the species in the region can be calculated from these areas and the population densities, as described above. When this is done for all yields in the permissible range, the yield at which the highest total population occurs can be obtained. This is referred to as the optimal yield on farmed land for the species, conditional on the production target.

#### Determining best farming strategy for biodiversity

**Figure 2:**
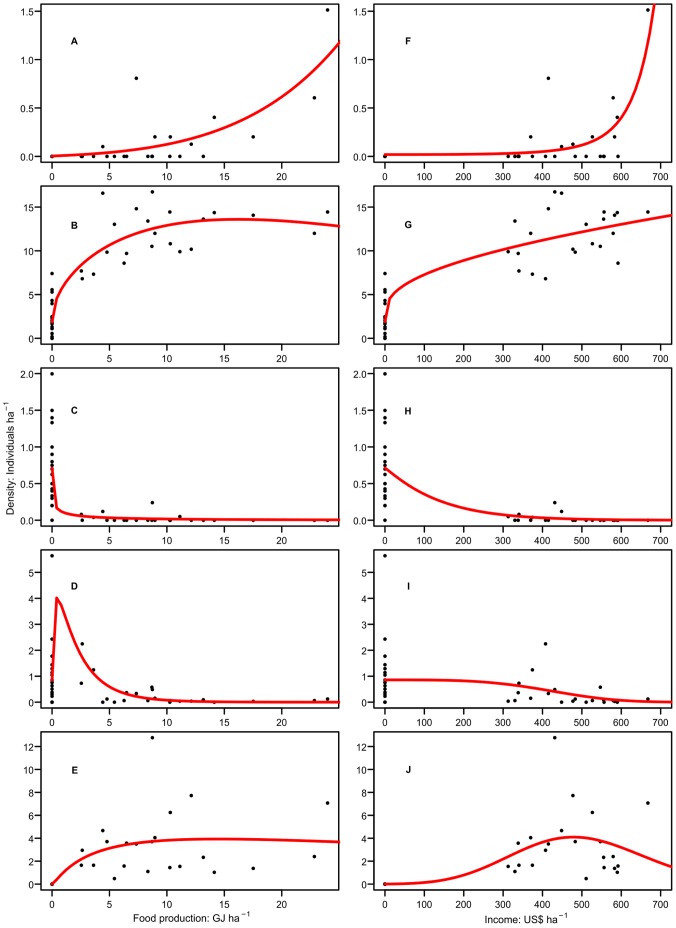
Examples of species with different types of fitted density-yield functions. (A, F) Cattle Egret *Bubulcus ibis*, which at all production targets is a winner for which land sparing is the best strategy. (B, G) Common Bulbul *Pycnonotus barbatus*, a winner for which land sharing is always the best strategy. (C, H) Black-necked Weaver *Ploceus nigricollis*, a loser for which land sparing is always the best strategy. (D, I) Splendid-glossy Starling *Lamprotornis splendidus*, a loser for which the best strategy depends on the production target. (E, J) Black-headed Weaver *Ploceus melanocephalus*, a winner for which the best strategy depends on the production target.

At a given production target and for species whose optimal yield was the lowest permissible yield, land sharing with low-yield farming would be the strategy under which those species would have the largest total population, which we call the best strategy. For species whose optimal yield was the highest permissible yield, land sparing with high-yield farming would be the best strategy. For species whose optimal yield was neither the lowest permissible yield nor the highest permissible yield, an intermediate yield would be best. For a given production target, species were classified as doing best with land sharing and low yields, land sparing and high yields or some intermediate strategy. At each production target all bird species were also classified as winners or losers in relation to agriculture according to whether their total population size would be higher or lower than the baseline if there was any farmed land within the study region. Winners were those species for which the total population size in the province was always equal to, or larger than, the baseline population, regardless of the yield of farming within the permissible range. Losers were those species with total populations lower than the baseline population at some (or all) permissible yields. Winners would be expected always to have more favourable conservation status than the average state for the distant past because their population is higher than the baseline population, regardless of production target and yield. Losers are species whose total population could potentially fall below the baseline as a result of agriculture, and therefore their conservation status is more sensitive to choices made about land allocation to farming at different yields. Our definition of losers includes both species which always decline as agricultural production increases, and others which have higher populations than the baseline at some yields, but lower populations at others. Figure 2 shows example density-yield curves for winners and losers. We calculated the optimal strategy for each species at production targets ranging from that equivalent to producing a single unit (1 GJ or $1 US) of output per hectare over the entire region, to the equivalent of farming the entire region at the maximum value of observed yield in any of our study sites.

#### Global range size

We compared the proportion of species that were winners and losers and with different optimal strategies between species with large global range sizes and those with smaller global range sizes, which are often those of greater conservation concern. Range sizes were obtained as the Extent of Occurrence (EOO) given by the World Bird Database (URL: http://www.birdlife.org/datazone/species/search: accessed on 27/10/2011). Species with an EOO of greater than 3,000,000 km^2^ were classified as having a large range size with those below having a small range size (see [4]). In the 26 cases where no extent of occurrence was for given breeding range other sources were referred to [32], [33]. This resulted in 91 species having small ranges and 165 species having large ranges.

#### Sample sizes

Since many species of greater conservation concern are likely to be found at low densities we wished to avoid biasing our conclusions by removing those species with low sample sizes. However, such species are likely to have less precisely estimated density-yield functions and this might have undue influence on the frequencies of different types of density-yield curves. In order to gauge the effect of retaining or excluding rare species, we compared our conclusions when species with fewer than 30 records were excluded with conclusions based upon all species.

## Results

The mean population density of each species in forest and farmland is shown in Table S2 and coefficients of the fitted density-yield functions are given in Table S3 for both the food energy and income models.

**Figure 3:**
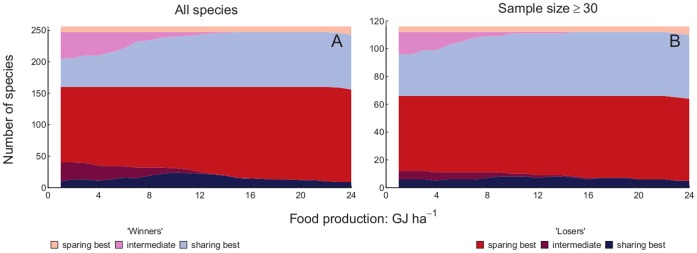
Winners and losers with food energy production targets by sample size. Number of species which have larger total populations with than without agriculture (winners: light colours) and those with smaller total populations (losers: dark colours) in relation to the production target for food energy. Species which have their largest total populations with the highest energy yield and land sparing (red/pink) those with largest populations with lowest permissible energy yield (land sharing: dark/light blue) and those benefitting most from intermediate yield (dark/light purple) are shown separately Maximum permissible yield was 30 GJ ha^−1^ year^−1^, 1.25 times the maximum observed yield. A is for all species, B is for species with a sample size of 30 individuals or greater.

**Figure 4:**
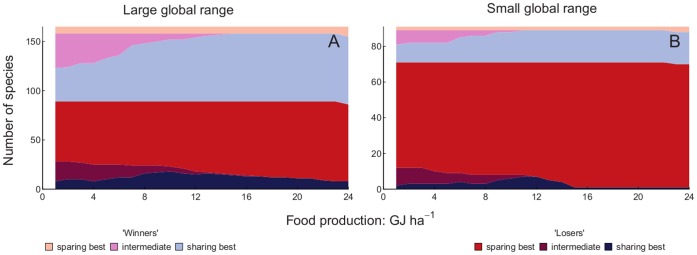
Winners and losers with food energy production targets by range size. Number of species which have larger total populations with than without agriculture (winners: light colours) and those with smaller total populations (losers: dark colours) in relation to the production target for food energy. Species which have their largest total populations with the highest energy yield and land sparing (red/pink) those with largest populations with lowest permissible energy yield (land sharing: dark/light blue) and those benefitting most from intermediate yield (dark/light purple) are shown separately. Conventions are as for Figure 3. A is for species with a large global range, B is for species with a small global range.

**Figure 5:**
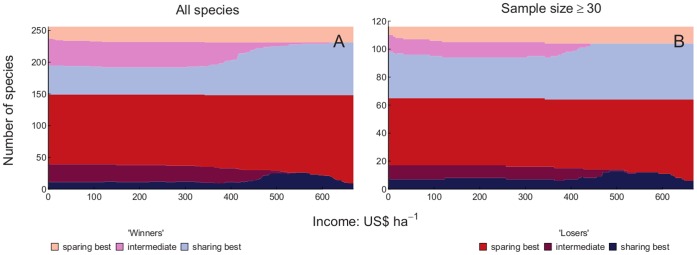
Winners and losers with gross income production targets by sample size. Number of species which have larger total populations with than without agriculture (winners: light colours) and those with smaller total populations (losers: dark colours) in relation to the production target for income. Species which have their largest total populations with the highest income and land sparing (red/pink) those with largest populations with lowest permissible income (land sharing: dark/light blue) and those benefitting most from intermediate yield (dark/light purple) are shown separately. Maximum permissible income was 835 US$ ha^−1^ year^−1^, 1.25 times the maximum observed yield. A is for all species, B is for species with a sample size of 30 individuals or greater.

**Figure 6:**
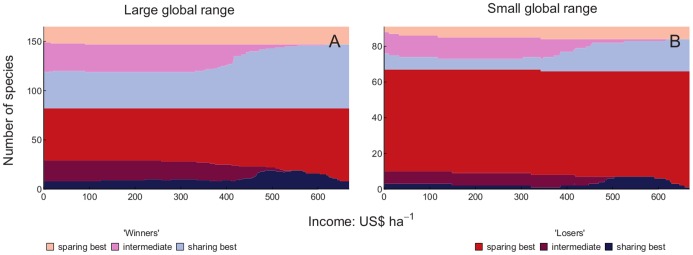
Winners and losers with gross income production targets by range size. Number of species which have larger total populations with than without agriculture (winners: light colours) and those with smaller total populations (losers: dark colours) in relation to the production target for income. Species which have their largest total populations with the highest income yield and land sparing (red/pink) those with largest populations with lowest permissible income yield (land sharing: dark/light blue) and those benefitting most from intermediate yield (dark/light purple) are shown separately. Conventions are as in Figure 5. A is for species with a large global range, B is for species with a small global range.

The numbers of winner and loser species with each of three categories of optimal yield (high yield, intermediate yield and low yield) were plotted against production target. We included all species when drawing conclusions based upon Figure 3, Figure 4, Figure 5 and Figure 6, since for both measures of yield removing species with 30 or fewer records did not alter the results markedly (Figures 3 and 5). More species were losers than were winners. Land sparing gave higher total populations of more of the loser species than did land sharing at all production targets, but the proportion of loser species doing best with land sparing increased as the production target increased. Land sharing gave higher total populations of more of the winner species than did land sparing at all production targets. The proportion of winner species doing best with land sharing increased as the production target increased. Ignoring whether species were winners or losers, there was an overall majority of species that would benefit from land sparing compared with species that would benefit from land sharing (Figures 3A and 5A). Intermediate yields were best for a relatively small proportion of species for both winners and losers and that proportion decreased markedly with increasing production target. The proportion of loser species was higher for species with small than large global ranges (Figures 4 and 6). For both losers and winners the proportion of species doing best with land sparing rather than land sharing was higher for small range than large range species (Figures 4 and 6).

## Discussion

Our results are remarkably consistent with those for birds and trees in Ghana and India [4], where land sparing with high-yield farming was estimated to give the largest total population size for most species of both groups, this finding being robust to the use of two different measures of yield. This was especially the case when species expected to have lower total populations than the baseline when agriculture is present (losers) are considered, but was also the case overall. Indeed, the Uganda models predict an even greater proportion of bird species would benefit from land sparing in comparison to land sharing than in Ghana and India. The greater preponderance of species that benefit from land sparing among losers than winners is considered to be of relevance to conservation because we think it likely that winner species tolerant of open habitats on farmland are likely to have larger populations under current conditions than at most times during their evolutionary history [34]. Hence, we expect loser species to be at higher current and future risk of adverse conservation status than winners, and therefore the response of losers to farming yield to be of greater conservation significance than that of winners. The greater representation of losers amongst species with smaller global ranges is also consistent with results for both birds and trees in Ghana and India. Small geographical range size is currently the single best predictor of threat of extinction in terrestrial bird species [35], so this information on the degree to which species in the two range size groups tolerate farming is of relevance to their future conservation. Although all species included in this analysis are classified globally as Least Concern by the IUCN red list (URL: http://www.iucnredlist.org/: accessed on 27/10/2011), deforestation and agricultural encroachment are continuing and likely to affect some of these species in future. Species tend to have much more restricted ranges than is indicated by their Extent of Occurrence [35]. Whilst the yields presented here were based, of necessity, on interviews with farmers who relied mostly on memory, the similarities between this and previous studies suggest that the yield data was sound and represented a true gradient of production intensity.

The forest fragments in our survey were mostly small and bird species that prefer forest interiors might be absent or at low density. A targeted survey of the largest and best quality forest sites in the region (probably only possible in Mabira and Dimmo) might produce higher population densities in forests (with zero agricultural yield) for these interior species. This would make our findings about the predominance of loser species for which land sparing is optimal conservative. Recent research in Uganda has suggested that forest birds move among forest fragments to a greater extent than was previously thought [25], so isolated small forests are still likely to have conservation value. Recent deforestration trends in Africa are such that they predict substantial forest loss over the next 50 years [24] and it is also possible that there is a lag effect in the response of bird density to recent deforestation. This might cause density-yield relationships to change somewhat over time.

Of 256 species 10 were Palearctic migrants, wintering in Africa between September and May [22], totalling 31 registrations (Table S2) of which Wood Warbler *Phylloscopus sibilatrix* was detected in forest only and Willow Warbler *Phylloscopus trochilus* was detected in forest and farmland with the remainder in farmland only, consistent with expected habitat requirements [22]. This indicates that the forest surveys were sufficient to register use of forest habitat by migrants between February and April. The period during which migrants are on breeding grounds will not have affected the density-yield relationships for those occurring in one habitat only. Partial intra-African migrants totalled 10 species, all of which are recorded in southern Uganda during the period of the forest surveys [22]. Two were observed only in forest and five only in farmland, Broad-billed Roller *Eurystomus glaucurus* and White-throated Bee-eater *Merops albicollis* were detected only rarely in forest (4 out of 22 registrations and 23 out of 484 registrations respectively), all of which is consistent with the expected habitat requirements of these nine species [22], so density-yield relationships are unlikely to have been biased. Red-chested Cuckoo *Cuculus solitaries* was recorded 24 times in forest and 27 times in farmland but the fitted model (Table S3) is consistent with what might be expected of this forest generalist [22]. Certain species might have seasonally-variable detectability due to changes in behaviour, vocalisations or vegetation but surveys were conducted throughout the year in farmland so maximising the chance that relative occurrence at each site will have been recorded. We have no reason to suspect that, other than the movements of potential migrants discussed above, species change their habitat use at particular times during the year [22].

Our results suggest that, at least within this tropical forested landscape, bird conservation would be best served by maintaining as much natural habitat as possible. This could benefit forest specialist species with small ranges which were observed only in our forest sites, such as, Weyns’s Weaver *Ploceus weynsi* and Joyful Greenbul *Chlorocichla laetissima* as well as, potentially, forest specialists with larger ranges such as Yellow-spotted Barbet *Buccanodon duchaillui*. In our study sites small range, more generalist species, such as Black-necked Weaver *Ploceus nigricollis* and Black-headed Weaver *Ploceus melanocephalus*, which both occurred on farmland (Figure 2), may, potentially, benefit from land sharing. There are examples, particularly from temperate regions (e.g. [36]) of some bird species being dependent on farmland habitat. Recently, dependency on low-intensity farming has also been claimed for some globally-threatened bird species in developing countries [37]. Any land sparing initiative aiming to increase yields on farmland should avoid doing so on farmland important for such species.

There has been criticism of the conclusions from Ghana and India [4] for paying insufficient attention to social and ecological complexities [38]. However, the approach taken here is not intended to provide detailed prescriptions for future landscape change, nor to address all of the complex issues involved in land-use change. Instead, it aims to test the widespread assumption that encouragement of low-yielding farmlands is necessarily the best option for conservation, using species-level and yield data for birds in Uganda that are more detailed than have been collected previously.

There are several aspects which the model described here does not take into account, such as social complexities and ecosystem services. To address these aspects will require further information on trade-offs and synergies between socio-economic and service outcomes and the biodiversity outcomes we have described, including quantification of the reliance of agriculture on ecosystem services [34], [39], [40]. Studies to collect such information should address the flaws in sampling design, inappropriate metrics, and/or failure to measure biodiversity baselines that have undermined the conclusions of many previous studies [34], [41].

A further important concern is to identify the social and governance contexts in which increasing yields might be effective as part of a strategy to protect natural habitats. There is good evidence that yield-increasing technologies can increase rather than decrease habitat conversion at local scales [11], [42], and at larger scales the evidence for sparing without any explicit policies to deliver it is weak [43], [44]. However, land sharing interventions are also often ineffective in practice [14], [45], and it seems premature to dismiss land sparing as a strategy when policy interventions specifically designed to achieve it have not yet been designed and tested. To help ensure that decision-makers, whether government bodies or local community leaders, take biodiversity into account it is imperative to integrate biodiversity conservation into policies and decision frameworks for resource production and consumption [44], [46], [47].

### Conclusion

Despite the close agreement between our results and those from Ghana and India [4], there are reasons to remain cautious about generalising our conclusion that land sparing has greater potential biodiversity benefits than land sharing. Studies are needed in more regions. In addition, further work is needed to understand how our conclusions might be affected by the inclusion of other objectives (such as social objectives), the spatial configuration of land uses, and the social or political feasibility of implementing particular strategies. However, we can draw some firm conclusions. None of the farming systems we examined in the banana-coffee arc around Lake Victoria are a substitute for relatively intact forests. We suggest that conservationists should avoid the promotion of low-yield farming where that is likely to result in further expansion into forests, unless a quantitative study on likely impacts on species’ populations indicates that this will be beneficial. Instead, we suggest that they explore the potential of linked policies to deliver land sparing, for example by directing development aid towards small farmers to increase yields on existing farmland, within a land-use planning framework (at regional or community level) which limits expansion of farmland into forests.

## Supporting Information

Table S1 Mean difference in total bird registrations and species richness between preliminary and main survey visits. N = number of site pairs (sites where a species was absent on both visits are not included).
(DOCX)

Table S2 Population densities (individuals ha^−1^) of all bird species, estimated for forest and farmland by sum(n)/sum(v) across all sites in each habitat, where *n* is the number of individuals of that species recorded, *v* = (*a* × *e*), where *a* is the effective detection area per survey point from Distance and *e* is effort (number of point counts conducted at the site). Note that *a* differed between farmland and forest sites for those species for which a habitat-specific detection function was used in program Distance.
(DOCX)

Table S3 Maximum-likelihood estimates of the coefficients for density-yield models for each species. Density is expressed as individuals ha^−1^, yield in food energy, GJ ha^−1^ year^−1^ and gross income, US$ ha^−1^ year^−1^. Where species were observed only in forest b_0_ was set at the natural logarithm of the calculated density in forest and zeroes are given for the other model parameters. Scientific names are given in Table S2.
(DOCX)

Text S1 The effect of a settling period on the number of individuals seen in the ten minute point count period at forest sites.
(DOCX)
