# Supplementary material for: Conserving the Birds of Uganda’s Banana-Coffee Arc: Land Sparing and Land Sharing Compared
Source: PLoS One. 2013 Feb 4;8(2):e54597. doi: 10.1371/journal.pone.0054597 (PMC3563584; doi:10.1371/journal.pone.0054597)
Supplement: Table S1 — Mean difference in total bird registrations and species richness between preliminary and main survey visits. N = number of site pairs (sites where a species was absent on both visits are not included). (DOCX) [file pone.0054597.s001.docx]

Hulme et al. Supplementary material Table S1. Mean difference in total bird registrations and species richness between preliminary and main survey visits. N = number of site pairs (sites where a species was absent on both visits are not included).

| Species | Mean difference | se | n | T | P |
| --- | --- | --- | --- | --- | --- |
| Eastern-grey Plantain-eater | 1.167 | 0.458 | 12 | 2.548 | 0.027 |
| Dusky Long-tailed Cuckoo | -0.444 | 0.176 | 9 | -2.530 | 0.035 |
| Black & White Casqued Hornbill | 1.000 | 0.458 | 15 | 2.185 | 0.046 |
| Western Nicator | -0.583 | 0.149 | 12 | -3.924 | 0.002 |
| Little Grey Greenbul | 0.714 | 0.286 | 7 | 2.500 | 0.047 |
| Green Crombec | -0.833 | 0.307 | 6 | -2.712 | 0.042 |
| Buff-throated Apalis | -3.429 | 1.417 | 14 | -2.420 | 0.031 |
| Purple-headed Starling | -2.700 | 1.146 | 10 | -2.357 | 0.043 |
|  |  |  |  |  |  |
