# Supplementary material for: Conserving the Birds of Uganda’s Banana-Coffee Arc: Land Sparing and Land Sharing Compared
Source: PLoS One. 2013 Feb 4;8(2):e54597. doi: 10.1371/journal.pone.0054597 (PMC3563584; doi:10.1371/journal.pone.0054597)
Supplement: Table S2 — Population densities (individuals ha−1) of all bird species, estimated for forest and farmland by sum(n)/sum(v) across all sites in each habitat, where n is the number of individuals of that species recorded, v = (a × e), where a is the effective detection area per survey point from Distance and e is effort (number of point counts conducted at the site). Note that a differed between farmland and forest sites for those species for which a habitat-specific detection function was used in program Distance. (DOCX) [file pone.0054597.s002.docx]

Hulme et al. Supplementary material Table S2. Population densities (individuals ha^-1^) of all bird species, estimated for forest and farmland by sum(n)/sum(v) across all sites in each habitat, where *n* is the number of individuals of that species recorded, *v* = (*a* × *e*), where *a* is the effective detection area per survey point from Distance and *e* is effort (number of point counts conducted at the site). Note that *a* differed between farmland and forest sites for those species for which a habitat-specific detection function was used in program Distance.

| Common Name | Scientific Name | Forest | | Farmland | |
| --- | --- | --- | --- | --- | --- |
|  |  | Count | Density  (Indiv ha^-1^) | Count | Density  (Indiv ha-^1^) |
| Scaly Francolin | *Francolinus squamatus* | 0 | 0 | 2 | 0.00605 |
| Red-necked Spurfowl | *Francolinus afer* | 0 | 0 | 6 | 0.0198 |
| Helmeted Guineafowl | *Numida meleagris* | 0 | 0 | 20 | 0.066 |
| Crested Guineafowl | *Guttera pucherani* | 1 | 0.00493 | 0 | 0 |
| Greater Honeyguide | *Indicator indicator* | 5 | 0.0506 | 0 | 0 |
| Lesser Honeyguide | *Indicator minor* | 7 | 0.0709 | 6 | 0.0311 |
| Least Honeyguide | *Indicator exilis* | 4 | 0.0319 | 0 | 0 |
| Cassin's Honeyguide | *Prodotiscus insignis* | 1 | 0.00772 | 0 | 0 |
| Nubian Woodpecker | *Campethera nubica* | 0 | 0 | 6 | 0.0126 |
| Buff-spotted Woodpecker | *Campethera nivosa* | 12 | 0.122 | 0 | 0 |
| Brown-eared Woodpecker | *Campethera caroli* | 12 | 0.088 | 0 | 0 |
| Golden-crowned Woodpecker | *Thripias xantholophus* | 33 | 0.263 | 0 | 0 |
| Grey Woodpecker | *Mesopicos goertae* | 0 | 0 | 2 | 0.0104 |
| Grey-throated Barbet | *Gymnobucco bonapartei* | 32 | 0.216 | 0 | 0 |
| Speckled Tinkerbird | *Pogoniulus scolopaceus* | 241 | 1.47 | 142 | 0.229 |
| Yellow-throated Tinkerbird | *Pogoniulus subsulphureus* | 71 | 0.401 | 2 | 0.00298 |
| Yellow-rumped Tinkerbird | *Pogoniulus bilineatus* | 69 | 0.309 | 165 | 0.503 |
| Yellow-fronted Tinkerbird | *Pogoniulus chrysoconus* | 5 | 0.0867 | 62 | 0.284 |
| Yellow-spotted Barbet | *Buccanodon duchaillui* | 44 | 0.525 | 0 | 0 |
| Hairy-breasted Barbet | *Tricholaema hirsuta* | 49 | 0.419 | 11 | 0.0248 |
| Spot-flanked Barbet | *Tricholaema lacrymosa* | 0 | 0 | 94 | 0.212 |
| White-headed Barbet | *Lybius leucocephalus* | 0 | 0 | 9 | 0.0243 |
| Double-toothed Barbet | *Lybius bidentatus* | 5 | 0.0418 | 85 | 0.187 |
| Yellow-billed Barbet | *Trachyphonus purpuratus* | 23 | 0.114 | 0 | 0 |
| Crowned Hornbill | *Tockus alboterminatus* | 56 | 0.308 | 52 | 0.0756 |
|  |  |  |  |  |  |
|  |  |  |  |  |  |
|  |  |  |  |  |  |
|  |  |  |  |  |  |
|  |  |  |  |  |  |
|  |  |  |  |  |  |
|  |  |  |  |  |  |
|  |  |  |  |  |  |
| Table S2 cont. |  |  |  |  |  |
| Common Name | Scientific Name | Forest | | Farmland | |
|  |  | Count | Density  (Indiv ha^-1^) | Count | Density  (Indiv ha-^1^) |
| African Pied Hornbill | *Tockus fasciatus* | 45 | 0.249 | 13 | 0.019 |
| Black-and-white-casqued Hornbill | *Bycanistes subcylindricus* | 172 | 1.47 | 68 | 0.154 |
| Green Woodhoopoe | *Phoeniculus purpureus* | 0 | 0 | 2 | 0.00541 |
| Forest Woodhoopoe | *Phoeniculus castaneiceps* | 13 | 0.0626 | 0 | 0 |
| Narina Trogon | *Apaloderma narina* | 39 | 0.188 | 0 | 0 |
| Lilac-breasted Roller | *Coracias caudatus* | 0 | 0 | 2 | 0.00548 |
| Broad-billed Roller | *Eurystomus glaucurus* | 4 | 0.0415 | 18 | 0.0493 |
| Blue-throated Roller | *Eurystomus gularis* | 1 | 0.00482 | 0 | 0 |
| White-bellied Kingfisher | *Alcedo leucogaster* | 1 | 0.00757 | 0 | 0 |
| African Pygmy-kingfisher | *Ceyx pictus* | 10 | 0.132 | 35 | 0.122 |
| African Dwarf-kingfisher | *Ceyx lecontei* | 5 | 0.0386 | 0 | 0 |
| Woodland Kingfisher | *Halcyon senegalensis* | 0 | 0 | 49 | 0.18 |
| Blue-breasted Kingfisher | *Halcyon malimbica* | 30 | 0.144 | 0 | 0 |
| Striped Kingfisher | *Halcyon chelicuti* | 0 | 0 | 27 | 0.074 |
| Pied Kingfisher | *Ceryle rudis* | 0 | 0 | 7 | 0.0224 |
| Little Bee-eater | *Merops pusillus* | 0 | 0 | 4 | 0.0135 |
| White-throated Bee-eater | *Merops albicollis* | 23 | 0.429 | 484 | 1.12 |
| Blue-cheeked Bee-eater | *Merops persicus* | 0 | 0 | 1 | 0.00274 |
| European Bee-eater | *Merops apiaster* | 0 | 0 | 5 | 0.0137 |
| Speckled Mousebird | *Colius striatus* | 0 | 0 | 323 | 1.25 |
| Blue-naped Mousebird | *Urocolius macrourus* | 0 | 0 | 1 | 0.00183 |
| Levaillant's Cuckoo | *Clamator levaillantii* | 0 | 0 | 8 | 0.0261 |
| Red-chested Cuckoo | *Cuculus solitarius* | 24 | 0.15 | 27 | 0.0446 |
| Black Cuckoo | *Cuculus clamosus* | 9 | 0.0695 | 0 | 0 |
| Dusky Long-tailed Cuckoo | *Cercococcyx mechowi* | 46 | 0.206 | 0 | 0 |
| Klaas's Cuckoo | *Chrysococcyx klaas* | 10 | 0.066 | 35 | 0.061 |
| African Emerald Cuckoo | *Chrysococcyx cupreus* | 10 | 0.0482 | 20 | 0.0419 |
| Didric Cuckoo | *Chrysococcyx caprius* | 0 | 0 | 35 | 0.0734 |
| Yellowbill | *Ceuthmochares aereus* | 54 | 1.1 | 0 | 0 |
| White-browed Coucal | *Centropus superciliosus* | 0 | 0 | 65 | 0.258 |
| Grey Parrot | *Psittacus erithacus* | 7 | 0.0473 | 4 | 0.00939 |
| Meyer's Parrot | *Poicephalus meyeri* | 0 | 0 | 64 | 0.0903 |
| Red-headed Lovebird | *Agapornis pullarius* | 2 | 0.0282 | 41 | 0.153 |
| Black-billed Turaco | *Tauraco schuetti* | 4 | 0.027 | 0 | 0 |
| Ross's Turaco | *Musophaga rossae* | 11 | 0.0738 | 74 | 0.131 |
| Bare-faced Go-away-bird | *Corythaixoides personatus* | 0 | 0 | 3 | 0.00704 |
| Eastern Grey Plantain-eater | *Crinifer zonurus* | 41 | 0.631 | 735 | 1.83 |
| Great Blue Turaco | *Corythaeola cristata* | 80 | 0.382 | 178 | 0.541 |
| African Wood-owl | *Strix woodfordii* | 3 | 0.0363 | 0 | 0 |
| Afep Pigeon | *Columba unicincta* | 13 | 0.0647 | 0 | 0 |
| Feral Pigeon | *Columba livia* | 0 | 0 | 15 | 0.0377 |
|  |  |  |  |  |  |
|  |  |  |  |  |  |
|  |  |  |  |  |  |
| Table S2 cont. |  |  |  |  |  |
| Common Name | Scientific Name | Forest | | Farmland | |
|  |  | Count | Density  (Indiv ha^-1^) | Count | Density  (Indiv ha-^1^) |
| Laughing Dove | *Stigmatopelia senegalensis* | 0 | 0 | 48 | 0.132 |
| Red-eyed Dove | *Streptopelia semitorquata* | 17 | 0.181 | 422 | 1.18 |
| Blue-spotted Wood-dove | *Turtur afer* | 10 | 0.0821 | 213 | 0.461 |
| Tambourine Dove | *Turtur tympanistria* | 72 | 0.322 | 104 | 0.228 |
| African Green-pigeon | *Treron calvus* | 22 | 0.175 | 112 | 0.235 |
| White-spotted Flufftail | *Sarothrura pulchra* | 45 | 0.201 | 0 | 0 |
| Wattled Lapwing | *Vanellus senegallus* | 0 | 0 | 173 | 0.643 |
| Osprey | *Pandion haliaetus* | 0 | 0 | 1 | 0.0032 |
| Black-winged Kite | *Elanus caeruleus* | 0 | 0 | 4 | 0.0128 |
| Black Kite | *Milvus migrans* | 0 | 0 | 35 | 0.112 |
| African Fish-eagle | *Haliaeetus vocifer* | 0 | 0 | 4 | 0.0128 |
| Palm-nut Vulture | *Gypohierax angolensis* | 0 | 0 | 1 | 0.00244 |
| Hooded Vulture | *Necrosyrtes monachus* | 0 | 0 | 2 | 0.00639 |
| Brown Snake-eagle | *Circaetus cinereus* | 0 | 0 | 8 | 0.0256 |
| African Marsh-harrier | *Circus ranivorus* | 0 | 0 | 1 | 0.0032 |
| African Harrier-hawk | *Polyboroides typus* | 3 | 0.0363 | 10 | 0.032 |
| Lizard Buzzard | *Kaupifalco monogrammicus* | 3 | 0.04 | 169 | 0.594 |
| Gabar Goshawk | *Melierax gabar* | 0 | 0 | 2 | 0.00639 |
| African Goshawk | *Accipiter tachiro* | 1 | 0.0121 | 0 | 0 |
| Shikra | *Accipiter badius* | 0 | 0 | 3 | 0.00959 |
| Little Sparrowhawk | *Accipiter minullus* | 1 | 0.0121 | 1 | 0.0032 |
| Black Goshawk | *Accipiter melanoleucus* | 16 | 0.194 | 0 | 0 |
| Wahlberg's Eagle | *Aquila wahlbergi* | 0 | 0 | 6 | 0.0192 |
| African Hawk-eagle | *Hieraaetus spilogaster* | 0 | 0 | 2 | 0.00639 |
| Long-crested Eagle | *Lophaetus occipitalis* | 2 | 0.0242 | 5 | 0.016 |
| Crowned Hawk-eagle | *Stephanoaetus coronatus* | 2 | 0.0242 | 0 | 0 |
| Black-headed Heron | *Ardea melanocephala* | 0 | 0 | 16 | 0.0528 |
| Cattle Egret | *Bubulcus ibis* | 0 | 0 | 41 | 0.191 |
| Hamerkop | *Scopus umbretta* | 0 | 0 | 3 | 0.0099 |
| Hadada Ibis | *Bostrychia hagedash* | 0 | 0 | 36 | 0.119 |
| Marabou Stork | *Leptoptilos crumeniferus* | 0 | 0 | 6 | 0.0192 |
| Grey-backed Fiscal | *Lanius excubitoroides* | 0 | 0 | 20 | 0.0548 |
| Common Fiscal | *Lanius collaris* | 0 | 0 | 1 | 0.00274 |
| Western Black-headed Oriole | *Oriolus brachyrhynchus* | 31 | 0.229 | 0 | 0 |
| African Black-headed Oriole | *Oriolus larvatus* | 0 | 0 | 27 | 0.0704 |
| Fork-tailed Drongo | *Dicrurus adsimilis* | 0 | 0 | 21 | 0.109 |
| Velvet-mantled Drongo | *Dicrurus adsimilis modestus* | 22 | 0.175 | 0 | 0 |
| Pied Crow | *Corvus albus* | 0 | 0 | 8 | 0.021 |
| Black Cuckooshrike | *Campephaga flava* | 6 | 0.0608 | 0 | 0 |
|  |  |  |  |  |  |
|  |  |  |  |  |  |
|  |  |  |  |  |  |
| Table S2 cont. |  |  |  |  |  |
| Common Name | Scientific Name | Forest | | Farmland | |
|  |  | Count | Density  (Indiv ha^-1^) | Count | Density  (Indiv ha-^1^) |
| Red-shouldered Cuckooshrike | *Campephaga phoenicea* | 0 | 0 | 1 | 0.00518 |
| Purple-throated Cuckooshrike | *Campephaga quiscalina* | 8 | 0.0637 | 0 | 0 |
| African Blue-flycatcher | *Elminia longicauda* | 11 | 0.28 | 173 | 1.16 |
| Dusky Crested-flycatcher | *Elminia nigromitrata* | 11 | 0.0807 | 0 | 0 |
| Blue-headed Crested-flycatcher | *Trochocercus nitens* | 2 | 0.0147 | 0 | 0 |
| African Paradise-flycatcher | *Terpsiphone viridis* | 0 | 0 | 172 | 0.342 |
| Black-headed Paradise-flycatcher | *Terpsiphone rufiventer* | 151 | 0.961 | 0 | 0 |
| Northern Puffback | *Dryoscopus gambensis* | 5 | 0.0415 | 62 | 0.136 |
| Black-crowned Tchagra | *Tchagra senegalus* | 0 | 0 | 6 | 0.0195 |
| Brown-crowned Tchagra | *Tchagra australis* | 0 | 0 | 63 | 0.15 |
| Ethiopian Boubou | *Laniarius aethiopicus* | 0 | 0 | 63 | 0.0743 |
| Black-headed Gonolek | *Laniarius erythrogaster* | 0 | 0 | 8 | 0.0242 |
| Sooty Boubou | *Laniarius leucorhynchus* | 10 | 0.102 | 0 | 0 |
| Grey-green Bush-shrike | *Telophorus bocagei* | 1 | 0.0131 | 1 | 0.00345 |
| African Shrike-flycatcher | *Megabyas flammulatus* | 16 | 0.117 | 0 | 0 |
| Black-and-white Shrike-flycatche | *Bias musicus* | 25 | 0.138 | 76 | 0.111 |
| Brown-throated Wattle-eye | *Platysteira cyanea* | 19 | 0.252 | 111 | 0.388 |
| Chestnut Wattle-eye | *Platysteira castanea* | 51 | 0.365 | 0 | 0 |
| Jameson's Wattle-eye | *Platysteira jamesoni* | 10 | 0.0734 | 0 | 0 |
| Rufous Flycatcher-thrush | *Stizorhina fraseri* | 59 | 0.27 | 0 | 0 |
| African Thrush | *Turdus pelios* | 8 | 0.0789 | 422 | 1.1 |
| Brown-chested Alethe | *Alethe poliocephala* | 11 | 0.0543 | 0 | 0 |
| White-tailed Alethe | *Alethe diademata* | 42 | 0.188 | 0 | 0 |
| Northern Black Flycatcher | *Melaenornis edolioides* | 2 | 0.0262 | 75 | 0.259 |
| African Dusky Flycatcher | *Muscicapa adusta* | 1 | 0.00772 | 24 | 0.0808 |
| Dusky-blue Flycatcher | *Muscicapa comitata* | 1 | 0.00772 | 0 | 0 |
| Ashy Flycatcher | *Muscicapa caerulescens* | 22 | 0.228 | 2 | 0.00548 |
| Grey-throated Tit-flycatcher | *Myioparus griseigularis* | 23 | 0.174 | 0 | 0 |
| Grey Tit-flycatcher | *Myioparus plumbeus* | 8 | 0.0831 | 0 | 0 |
| Blue-shouldered Robin-chat | *Cossypha cyanocampter* | 25 | 0.254 | 0 | 0 |
| White-browed Robin-chat | *Cossypha heuglini* | 0 | 0 | 35 | 0.121 |
| Red-capped Robin-chat | *Cossypha natalensis* | 7 | 0.0711 | 0 | 0 |
| Snowy-crowned Robin-chat | *Cossypha niveicapilla* | 9 | 0.0914 | 8 | 0.00943 |
| Brown-backed Scrub Robin | *Erythropygia hartlaubi* | 0 | 0 | 35 | 0.103 |
| Red-backed Scrub-robin | *Erythropygia leucophrys* | 0 | 0 | 8 | 0.0276 |
| Forest Robin | *Stiphrornis erythrothorax* | 28 | 0.138 | 0 | 0 |
| Whinchat | *Saxicola rubetra* | 0 | 0 | 4 | 0.0105 |
| Sooty Chat | *Myrmecocichla nigra* | 0 | 0 | 31 | 0.102 |
| Chestnut-winged Starling | *Onychognathus fulgidus* | 1 | 0.00676 | 0 | 0 |
|  |  |  |  |  |  |
|  |  |  |  |  |  |
|  |  |  |  |  |  |
| Table S2 cont. |  |  |  |  |  |
| Common Name | Scientific Name | Forest | | Farmland | |
|  |  | Count | Density  (Indiv ha^-1^) | Count | Density  (Indiv ha-^1^) |
| Purple-headed Glossy-starling | *Lamprotornis purpureiceps* | 288 | 1.29 | 0 | 0 |
| Splendid Glossy-starling | *Lamprotornis splendidus* | 158 | 0.842 | 224 | 0.315 |
| Rueppell's Glossy-starling | *Lamprotornis purpuroptera* | 0 | 0 | 99 | 0.193 |
| Violet-backed Starling | *Cinnyricinclus leucogaster* | 30 | 0.369 | 10 | 0.0325 |
| African Penduline-tit | *Anthoscopus caroli* | 2 | 0.0261 | 0 | 0 |
| White-winged Tit | *Parus leucomelas* | 0 | 0 | 25 | 0.129 |
| White-winged Tit | *Parus leucomelas* | 2 | 0.0203 | 0 | 0 |
| Dusky Tit | *Parus funereus* | 6 | 0.044 | 0 | 0 |
| Common Bulbul | *Pycnonotus barbatus* | 143 | 1.86 | 3469 | 11.9 |
| Little Greenbul | *Andropadus virens* | 1054 | 10.1 | 357 | 0.907 |
| Grey Greenbul | *Andropadus gracilis* | 62 | 0.339 | 0 | 0 |
| Plain Greenbul | *Andropadus curvirostris* | 32 | 0.235 | 0 | 0 |
| Slender-billed Greenbul | *Andropadus gracilirostris* | 63 | 0.444 | 0 | 0 |
| Yellow-whiskered Greenbul | *Andropadus latirostris* | 118 | 0.716 | 0 | 0 |
| Honeyguide Greenbul | *Baeopogon indicator* | 7 | 0.0517 | 0 | 0 |
| Yellow-throated Greenbul | *Chlorocichla flavicollis* | 11 | 0.112 | 5 | 0.00589 |
| Joyful Greenbul | *Chlorocichla laetissima* | 6 | 0.0443 | 0 | 0 |
| Toro Olive Greenbul | *Phyllastrephus hypochloris* | 16 | 0.162 | 0 | 0 |
| White-throated Greenbul | *Phyllastrephus albigularis* | 126 | 0.945 | 0 | 0 |
| Common Bristlebill | *Bleda syndactylus* | 44 | 0.197 | 0 | 0 |
| Lesser Bristlebill | *Bleda notatus* | 40 | 0.179 | 0 | 0 |
| Yellow-spotted Nicator | *Nicator chloris* | 63 | 0.282 | 0 | 0 |
| Red-tailed Bulbul | *Criniger calurus* | 71 | 0.625 | 0 | 0 |
| Red-faced Cisticola | *Cisticola erythrops* | 0 | 0 | 391 | 0.715 |
| Winding Cisticola | *Cisticola galactotes* | 0 | 0 | 59 | 0.15 |
| Croaking Cisticola | *Cisticola natalensis* | 0 | 0 | 35 | 0.181 |
| Tawny-flanked Prinia | *Prinia subflava* | 0 | 0 | 1220 | 3.5 |
| White-chinned Prinia | *Prinia leucopogon* | 10 | 0.0493 | 4 | 0.0121 |
| Black-throated Apalis | *Apalis jacksoni* | 19 | 0.0937 | 0 | 0 |
| Buff-throated Apalis | *Apalis rufogularis* | 389 | 1.74 | 0 | 0 |
| Grey-capped Warbler | *Eminia lepida* | 0 | 0 | 1 | 0.002 |
| Green-backed Camaroptera | *Camaroptera brachyura* | 183 | 1.75 | 1498 | 5.6 |
| Yellow-browed Camaroptera | *Camaroptera superciliaris* | 35 | 0.173 | 0 | 0 |
| Olive-green Camaroptera | *Camaroptera chloronota* | 43 | 0.331 | 0 | 0 |
| African Yellow White-eye | *Zosterops senegalensis* | 79 | 1.82 | 601 | 3.65 |
| Black-faced Rufous Warbler | *Bathmocercus rufus* | 1 | 0.00734 | 0 | 0 |
| Moustached Grass-warbler | *Melocichla mentalis* | 0 | 0 | 10 | 0.0303 |
| Eastern Olivaceous Warbler | *Hippolais pallida* | 0 | 0 | 3 | 0.00908 |
| Icterine Warbler | *Hippolais icterina* | 0 | 0 | 4 | 0.0121 |
|  |  |  |  |  |  |
|  |  |  |  |  |  |
|  |  |  |  |  |  |
| Table S2 cont. |  |  |  |  |  |
| Common Name | Scientific Name | Forest | | Farmland | |
|  |  | Count | Density  (Indiv ha^-1^) | Count | Density  (Indiv ha-^1^) |
| Buff-bellied Warbler | *Phyllolais pulchella* | 0 | 0 | 1 | 0.00518 |
| Green Crombec | *Sylvietta virens* | 50 | 0.803 | 0 | 0 |
| Northern Crombec | *Sylvietta brachyura* | 1 | 0.00734 | 0 | 0 |
| Red-faced Crombec | *Sylvietta whytii* | 0 | 0 | 2 | 0.00651 |
| Yellow Longbill | *Macrosphenus flavicans* | 14 | 0.111 | 0 | 0 |
| Grey Longbill | *Macrosphenus concolor* | 13 | 0.104 | 0 | 0 |
| Green Hylia | *Hylia prasina* | 110 | 0.528 | 0 | 0 |
| Willow Warbler | *Phylloscopus trochilus* | 3 | 0.0304 | 3 | 0.0155 |
| Wood Warbler | *Phylloscopus sibilatrix* | 2 | 0.0203 | 0 | 0 |
| Scaly-breasted Illadopsis | *Illadopsis albipectus* | 16 | 0.0789 | 0 | 0 |
| Pale-breasted Illadopsis | *Illadopsis rufipennis* | 27 | 0.133 | 0 | 0 |
| Brown Illadopsis | *Illadopsis fulvescens* | 11 | 0.112 | 0 | 0 |
| Sharpe's Pied-babbler | *Turdoides sharpei* | 0 | 0 | 14 | 0.0424 |
| Brown Babbler | *Turdoides plebejus* | 0 | 0 | 1 | 0.00303 |
| Arrow-marked Babbler | *Turdoides jardineii* | 0 | 0 | 6 | 0.0182 |
| Garden Warbler | *Sylvia borin* | 0 | 0 | 2 | 0.00605 |
| Flappet Lark | *Mirafra rufocinnamomea* | 0 | 0 | 2 | 0.0066 |
| Scarlet-tufted Sunbird | *Anthreptes fraseri* | 18 | 0.227 | 0 | 0 |
| Green Sunbird | *Anthreptes rectirostris* | 18 | 0.207 | 0 | 0 |
| Collared Sunbird | *Anthreptes collaris* | 59 | 0.722 | 0 | 0 |
| Little Green Sunbird | *Nectarinia seimundi* | 71 | 1.03 | 0 | 0 |
| Olive Sunbird | *Nectarinia olivacea* | 110 | 1.47 | 0 | 0 |
| Green-headed Sunbird | *Nectarinia verticalis* | 45 | 0.556 | 45 | 0.255 |
| Blue-throated Brown Sunbird | *Nectarinia cyanolaema* | 38 | 0.438 | 0 | 0 |
| Green-throated Sunbird | *Nectarinia rubescens* | 19 | 0.104 | 0 | 0 |
| Scarlet-chested Sunbird | *Nectarinia senegalensis* | 12 | 0.157 | 608 | 2.1 |
| Variable Sunbird | *Nectarinia venusta* | 0 | 0 | 230 | 1.78 |
| Northern Double-collared Sunbird | *Nectarinia preussi* | 0 | 0 | 36 | 0.224 |
| Olive-bellied Sunbird | *Nectarinia chloropygia* | 7 | 0.183 | 102 | 0.705 |
| Copper Sunbird | *Nectarinia cuprea* | 0 | 0 | 46 | 0.275 |
| Bronze Sunbird | *Nectarinia kilimensis* | 0 | 0 | 1 | 0.00447 |
| Red-chested Sunbird | *Nectarinia erythrocerca* | 0 | 0 | 179 | 0.735 |
| Mariqua Sunbird | *Nectarinia mariquensis* | 0 | 0 | 33 | 0.147 |
| Superb Sunbird | *Nectarinia superba* | 9 | 0.104 | 0 | 0 |
| Northern Grey-headed Sparrow | *Passer griseus* | 0 | 0 | 918 | 3.28 |
| African Pied Wagtail | *Motacilla aguimp* | 0 | 0 | 24 | 0.0792 |
| Yellow Wagtail | *Motacilla flava* | 0 | 0 | 3 | 0.0099 |
| Yellow-throated Longclaw | *Macronyx croceus* | 0 | 0 | 18 | 0.0594 |
| Baglafecht Weaver | *Ploceus baglafecht* | 0 | 0 | 33 | 0.204 |
|  |  |  |  |  |  |
|  |  |  |  |  |  |
|  |  |  |  |  |  |
| Table S2 cont. |  |  |  |  |  |
| Common Name | Scientific Name | Forest | | Farmland | |
|  |  | Count | Density  (Indiv ha^-1^) | Count | Density  (Indiv ha-^1^) |
| Spectacled Weaver | *Ploceus ocularis* | 0 | 0 | 3 | 0.0103 |
| Black-necked Weaver | *Ploceus nigricollis* | 102 | 0.715 | 13 | 0.024 |
| Vieillot's Black Weaver | *Ploceus nigerrimus* | 39 | 0.174 | 96 | 0.281 |
| Weyns's Weaver | *Ploceus weynsi* | 1442 | 11.6 | 0 | 0 |
| Black-headed Weaver | *Ploceus melanocephalus* | 0 | 0 | 1088 | 3.46 |
| Golden-backed Weaver | *Ploceus jacksoni* | 0 | 0 | 3 | 0.0164 |
| Yellow-mantled Weaver | *Ploceus tricolor* | 31 | 0.229 | 0 | 0 |
| Red-headed Malimbe | *Malimbus rubricollis* | 14 | 0.111 | 1 | 0.0021 |
| Black Bishop | *Euplectes gierowii* | 0 | 0 | 2 | 0.0109 |
| Black-winged Bishop | *Euplectes hordeaceus* | 0 | 0 | 1 | 0.00262 |
| Fan-tailed Widowbird | *Euplectes axillaris* | 0 | 0 | 44 | 0.0912 |
| Yellow-shouldered Widowbird | *Euplectes macroura* | 0 | 0 | 1 | 0.00546 |
| Grosbeak Weaver | *Amblyospiza albifrons* | 24 | 0.273 | 3 | 0.0164 |
| White-breasted Negrofinch | *Nigrita fusconotus* | 34 | 0.251 | 0 | 0 |
| Grey-headed Negrofinch | *Nigrita canicapillus* | 39 | 0.25 | 7 | 0.0118 |
| Green-winged Pytilia | *Pytilia melba* | 0 | 0 | 8 | 0.0437 |
| Green-backed Twinspot | *Mandingoa nitidula* | 15 | 0.152 | 0 | 0 |
| Black-bellied Seedcracker | *Pyrenestes ostrinus* | 9 | 0.102 | 0 | 0 |
| Red-headed Bluebill | *Spermophaga ruficapilla* | 34 | 0.273 | 0 | 0 |
| Red-billed Firefinch | *Lagonosticta senegala* | 0 | 0 | 201 | 1.58 |
| African Firefinch | *Lagonosticta rubricata* | 0 | 0 | 10 | 0.0546 |
| Red-cheeked Cordonbleu | *Uraeginthus bengalus* | 0 | 0 | 145 | 1.03 |
| Crimson-rumped Waxbill | *Estrilda rhodopyga* | 0 | 0 | 10 | 0.0546 |
| Common Waxbill | *Estrilda astrild* | 0 | 0 | 12 | 0.042 |
| Black-crowned Waxbill | *Estrilda nonnula* | 0 | 0 | 380 | 3.51 |
| Bronze Munia | *Lonchura cucullata* | 0 | 0 | 2433 | 14.2 |
| Black-and-white Munia | *Lonchura bicolor* | 0 | 0 | 65 | 0.624 |
| Magpie Munia | *Lonchura fringilloides* | 3 | 0.0298 | 0 | 0 |
| Village Indigobird | *Vidua chalybeata* | 0 | 0 | 57 | 0.205 |
| Pin-tailed Whydah | *Vidua macroura* | 0 | 0 | 26 | 0.142 |
| Yellow-browed Citril | *Serinus frontalis* | 0 | 0 | 10 | 0.0546 |
| Black-throated Seedeater | *Serinus atrogularis* | 0 | 0 | 26 | 0.142 |
| Yellow-fronted Canary | *Serinus mozambicus* | 0 | 0 | 648 | 3.27 |
| African Golden-breasted Bunting | *Emberiza flaviventris* | 0 | 0 | 20 | 0.109 |
