# Supplementary material for: Conserving the Birds of Uganda’s Banana-Coffee Arc: Land Sparing and Land Sharing Compared
Source: PLoS One. 2013 Feb 4;8(2):e54597. doi: 10.1371/journal.pone.0054597 (PMC3563584; doi:10.1371/journal.pone.0054597)
Supplement: Table S3 — Maximum-likelihood estimates of the coefficients for density-yield models for each species. Density is expressed as individuals ha−1, yield in food energy, GJ ha−1 year−1 and gross income, US$ ha−1 year−1. Where species were observed only in forest b0 was set at the natural logarithm of the calculated density in forest and zeroes are given for the other model parameters. Scientific names are given in Table S2. (DOCX) [file pone.0054597.s003.docx]

Hulme et al. Supplementary material Table S3. Maximum-likelihood estimates of the coefficients for density-yield models for each species. Density is expressed as individuals ha^-1^, yield in food energy, GJ ha^-1^ year^-1^ and gross income, US$ ha^-1^ year^-1^. Where species were observed only in forest b_0_ was set at the natural logarithm of the calculated density in forest and zeroes are given for the other model parameters. Scientific names are given in Table S2.

| Common Name | Food Energy (GJ ha^-1^ year^-1^) | | | |  | Income (US$ ha^-1^ year^-1^) | | | |
| --- | --- | --- | --- | --- | --- | --- | --- | --- | --- |
|  | b0 | b1 | b2 | alpha |  | b0 | b1 | b2 | alpha |
| Scaly Francolin | -29 | 2.84 | -0.075 | 2.27 |  | -226 | 0.000951 | -1E-09 | 2.19 |
| Red-necked Spurfowl | -6.48 | 2.13 | -0.307 | 0.853 |  | -11.6 | 1.85E-06 | -9.3E-14 | 2.72 |
| Helmeted Guineafowl | -15.8 | 29.2 | -15.1 | 0.101 |  | -8.94 | 6.2 | 0 | 8.19E-04 |
| Crested Guineafowl | -4.57 | 0 | 0 | 0 |  | -4.57 | 0 | 0 | 0 |
| Greater Honeyguide | -2.38 | 0 | 0 | 0 |  | -2.38 | 0 | 0 | 0 |
| Lesser Honeyguide | -2.05 | 0.803 | -0.0757 | 2.45 |  | -2.68 | 5.67E-08 | -6.8E-16 | 3.1 |
| Least Honeyguide | -3.45 | 0 | 0 | 0 |  | -3.45 | 0 | 0 | 0 |
| Cassin's Honeyguide | -4.51 | 0 | 0 | 0 |  | -4.51 | 0 | 0 | 0 |
| Nubian Woodpecker | -11.5 | 15.1 | -6.77 | 0.224 |  | -7.43 | 1.34E-06 | -1.1E-13 | 2.58 |
| Buff-spotted Woodpecker | -1.5 | 0 | 0 | 0 |  | -1.5 | 0 | 0 | 0 |
| Brown-eared Woodpecker | -2.19 | 0 | 0 | 0 |  | -2.19 | 0 | 0 | 0 |
| Golden-crowned Woodpecker | -1.34 | 0 | 0 | 0 |  | -1.34 | 0 | 0 | 0 |
| Grey Woodpecker | -112 | 5.88 | -0.0782 | 1.81 |  | -220 | 0.000934 | -1E-09 | 2.16 |
| Grey-throated Barbet | -1.31 | 0 | 0 | 0 |  | -1.31 | 0 | 0 | 0 |
| Speckled Tinkerbird | 0.386 | -0.782 | 0.0684 | 0.665 |  | 0.389 | -7.2E-05 | 6.1E-10 | 1.76 |
| Yellow-throated Tinkerbird | -0.915 | -2.47 | 0 | 0.371 |  | -0.915 | -4.81 | 0 | 0.00312 |
| Yellow-rumped Tinkerbird | -0.964 | 0.0714 | -0.00535 | 0.881 |  | -0.986 | 0.000026 | 0 | 1.46 |
| Yellow-fronted Tinkerbird | -2.52 | 1.14 | -0.22 | 0.517 |  | -3.11 | 1.15E-07 | -1.3E-15 | 2.87 |
| Yellow-spotted Barbet | -2.32 | 0 | 0 | 0 |  | -2.32 | 0 | 0 | 0 |
| Hairy-breasted Barbet | -0.871 | -2.27 | 0 | 0.106 |  | -0.868 | -0.00162 | 0 | 1.23 |
|  |  |  |  |  |  |  |  |  |  |
| Table S3 cont. |  |  |  |  |  |  |  |  |  |
| Common Name | Food Energy (GJ ha^-1^ year^-1^) | | | |  | Income (US$ ha^-1^ year^-1^) | | | |
|  | b0 | b1 | b2 | alpha |  | b0 | b1 | b2 | alpha |
| Spot-flanked Barbet | -8.02 | 8.39 | -2.6 | 0.285 |  | -8.72 | 0.413 | -0.00575 | 0.602 |
| White-headed Barbet | -5.84 | 0.00016 | -1.7E-09 | 4.56 |  | -18.1 | 1.36E-06 | -2.9E-14 | 2.73 |
| Double-toothed Barbet | -3.24 | 0.851 | -0.0968 | 0.725 |  | -3.18 | 0.00013 | -2.3E-09 | 1.69 |
| Yellow-billed Barbet | -1.75 | 0 | 0 | 0 |  | -1.75 | 0 | 0 | 0 |
| Crowned Hornbill | -1.18 | -0.602 | 0 | 0.411 |  | -1.18 | -0.00305 | 0 | 1 |
| African Pied Hornbill | -1.39 | -2.57 | 0 | 6.22E-04 |  | -1.39 | -0.083 | 0 | 0.561 |
| Black-and-white-casqued Hornbill | 0.374 | -0.723 | 0 | 0.567 |  | 0.388 | -2.23 | 0 | 1.97E-03 |
| Green Woodhoopoe | -120 | 6.47 | -0.0888 | 1.79 |  | -213 | 0.000946 | -1.1E-09 | 2.16 |
| Forest Woodhoopoe | -2.49 | 0 | 0 | 0 |  | -2.49 | 0 | 0 | 0 |
| Narina Trogon | -1.39 | 0 | 0 | 0 |  | -1.39 | 0 | 0 | 0 |
| Lilac-breasted Roller | -112 | 5.74 | -0.0747 | 1.82 |  | -222 | 0.000897 | -9.2E-10 | 2.17 |
| Broad-billed Roller | -3.03 | -5E-06 | 2.55E-12 | 4.6 |  | -3.27 | 8.82E-14 | 0 | 4.6 |
| Blue-throated Roller | -5.06 | 0 | 0 | 0 |  | -5.06 | 0 | 0 | 0 |
| White-bellied Kingfisher | -4.88 | 0 | 0 | 0 |  | -4.88 | 0 | 0 | 0 |
| African Pygmy-kingfisher | -1.92 | -0.0355 | 0 | 0.815 |  | -1.78 | -1.7E-13 | 0 | 4.6 |
| African Dwarf-kingfisher | -2.9 | 0 | 0 | 0 |  | -2.9 | 0 | 0 | 0 |
| Woodland Kingfisher | -8.09 | 6.37 | 0 | 3.33E-04 |  | -11.1 | 15 | -4.82 | 0.134 |
| Blue-breasted Kingfisher | -1.66 | 0 | 0 | 0 |  | -1.66 | 0 | 0 | 0 |
| Striped Kingfisher | -258 | 481 | -226 | 4.98E-02 |  | -8.67 | 4.23 | -0.59 | 0.269 |
| Pied Kingfisher | -24.9 | 19.2 | 0 | 4.10E-02 |  | -9.71 | 5.89 | 0 | 6.16E-04 |
| Little Bee-eater | -47.1 | 2.05 | -0.023 | 1.87 |  | -215 | 0.000819 | -7.9E-10 | 2.27 |
| White-throated Bee-eater | -0.847 | 0.964 | 0 | 5.72E-05 |  | -0.788 | 0.0114 | 0 | 0.709 |
| Blue-cheeked Bee-eater | -233 | 8.97 | -0.0876 | 1.82 |  | -93.5 | 64.2 | 0 | 4.99E-02 |
|  |  |  |  |  |  |  |  |  |  |
| Table S3 cont. |  |  |  |  |  |  |  |  |  |
| Common Name | Food Energy (GJ ha^-1^ year^-1^) | | | |  | Income (US$ ha^-1^ year^-1^) | | | |
|  | b0 | b1 | b2 | alpha |  | b0 | b1 | b2 | alpha |
| European Bee-eater | -6.31 | 8.72E-05 | -4.9E-10 | 4.37 |  | -129 | 5.42 | -0.0583 | 0.611 |
| Speckled Mousebird | -529 | 960 | -435 | 4.99E-02 |  | -12.6 | 0.833 | -0.0133 | 0.566 |
| Blue-naped Mousebird | -185 | 9.72 | -0.13 | 1.8 |  | -157 | 0.000723 | -8.5E-10 | 2.15 |
| Levaillant's Cuckoo | -6.49 | 0.000105 | -5.5E-10 | 4.6 |  | -13 | 1.12E-06 | -2.9E-14 | 2.73 |
| Red-chested Cuckoo | -2.04 | -0.0518 | 0 | 1.4 |  | -1.89 | -1.6E-09 | 0 | 3.35 |
| Black Cuckoo | -2.32 | 0 | 0 | 0 |  | -2.32 | 0 | 0 | 0 |
| Dusky Long-tailed Cuckoo | -3.26 | 0 | 0 | 0 |  | -3.26 | 0 | 0 | 0 |
| Klaas's Cuckoo | -2.71 | -0.0598 | 0 | 0.188 |  | -2.85 | 2.85E-09 | 0 | 2.78 |
| African Emerald Cuckoo | -2.84 | -1.2E-05 | 0 | 4.6 |  | -3.03 | 0.00548 | -7.7E-06 | 1.08 |
| Didric Cuckoo | -150 | 288 | -140 | 4.96E-02 |  | -27.3 | 0.131 | -0.00017 | 0.976 |
| Yellowbill | -1.58 | 0 | 0 | 0 |  | -1.58 | 0 | 0 | 0 |
| White-browed Coucal | -349 | 635 | -290 | 5.02E-02 |  | -39 | 4.14 | -0.113 | 0.484 |
| Grey Parrot | -3.05 | -1.62 | 0 | 3.03E-05 |  | -3.09 | 6.57E-05 | -4.3E-10 | 2.06 |
| Meyer's Parrot | -7.85 | 3.28 | -0.461 | 0.517 |  | -11.9 | 12.4 | -3.4 | 0.153 |
| Red-headed Lovebird | -3.83 | 0.839 | -0.0687 | 0.954 |  | -5.21 | 2.68E-07 | -4.2E-15 | 2.8 |
| Black-billed Turaco | -3.39 | 0 | 0 | 0 |  | -3.39 | 0 | 0 | 0 |
| Ross's Turaco | -2.42 | 0.0362 | 0 | 0.979 |  | -2.63 | 0.00315 | 0 | 0.85 |
| Bare-faced Go-away-bird | -329 | 8.18 | -0.0511 | 1.86 |  | -192 | 0.000763 | -7.7E-10 | 2.09 |
| Eastern Grey Plantain-eater | -0.456 | 0.646 | 0 | 0.227 |  | -0.46 | 1.06 | 0 | 1.73E-04 |
| Great Blue Turaco | -0.629 | -5.4E-06 | 2.53E-12 | 4.6 |  | -0.653 | -3.4E-10 | 0 | 3.2 |
| African Wood-owl | -3.32 | 0 | 0 | 0 |  | -3.32 | 0 | 0 | 0 |
| Afep Pigeon | -2.32 | 0 | 0 | 0 |  | -2.32 | 0 | 0 | 0 |
| Feral Pigeon | -31.7 | 21.4 | -3.92 | 0.389 |  | -76.5 | 6.89 | -0.161 | 0.496 |
|  |  |  |  |  |  |  |  |  |  |
| Table S3 cont. |  |  |  |  |  |  |  |  |  |
| Common Name | Food Energy (GJ ha^-1^ year^-1^) | | | |  | Income (US$ ha^-1^ year^-1^) | | | |
|  | b0 | b1 | b2 | alpha |  | b0 | b1 | b2 | alpha |
| Laughing Dove | -589 | 1030 | -450 | 5.03E-02 |  | -22.8 | 15.3 | 0 | 4.94E-02 |
| Red-eyed Dove | -1.71 | 1.86 | 0 | 6.13E-03 |  | -1.71 | 1.87 | 0 | 1.07E-03 |
| Blue-spotted Wood-dove | -2.5 | 9.27 | -6.82 | 6.17E-02 |  | -2.5 | 1.41 | -0.217 | 0.259 |
| Tambourine Dove | -1.13 | -0.345 | 0 | 2.32E-04 |  | -1.02 | -2.6E-09 | 2.09E-18 | 3.22 |
| African Green-pigeon | -1.58 | 1.26E-06 | 0 | 4.16 |  | -1.74 | 0.288 | 0 | 4.12E-03 |
| White-spotted Flufftail | -3.28 | 0 | 0 | 0 |  | -3.28 | 0 | 0 | 0 |
| Wattled Lapwing | -7.76 | 24.4 | -16.4 | 3.13E-02 |  | -7.61 | 7 | 0 | 3.78E-03 |
| Osprey | -298 | 5.5 | -0.0256 | 1.82 |  | -107 | 74.7 | 0 | 4.95E-02 |
| Black-winged Kite | -10.6 | 6.22 | 0 | 7.55E-04 |  | -10.9 | 6.42 | 0 | 3.64E-03 |
| Black Kite | -40 | 33.6 | 0 | 4.92E-02 |  | -175 | 127 | 0 | 4.96E-02 |
| African Fish-eagle | -450 | 8.6 | -0.0411 | 1.8 |  | -216 | 0.000981 | -1.1E-09 | 2.05 |
| Palm-nut Vulture | -89.6 | 0.0142 | 0 | 2.74 |  | -197 | 6.86 | 0 | 0.514 |
| Hooded Vulture | -11.1 | 6.05 | 0 | 1.20E-03 |  | -34 | 2.07E-06 | -3.5E-14 | 2.74 |
| Brown Snake-eagle | -24.9 | 27.8 | -8.71 | 0.323 |  | -12.4 | 1.32E-06 | -4.3E-14 | 2.72 |
| African Marsh-harrier | -98.8 | 8.53 | -0.189 | 1.82 |  | -168 | 0.000731 | -8.1E-10 | 2.09 |
| African Harrier-hawk | -3.31 | -1.6E-05 | 7.68E-12 | 4.6 |  | -3.73 | 1.2E-13 | 0 | 4.6 |
| Lizard Buzzard | -3.25 | 1.7 | 0 | 0.211 |  | -3.22 | 1.69 | 0 | 7.59E-02 |
| Gabar Goshawk | -168 | 6.95 | -0.0728 | 1.93 |  | -216 | 0.000861 | -8.7E-10 | 2.17 |
| African Goshawk | -4.41 | 0 | 0 | 0 |  | -4.41 | 0 | 0 | 0 |
| Shikra | -8.76 | 11 | -5.01 | 0.308 |  | -16 | 2.29E-06 | -1E-13 | 2.72 |
| Little Sparrowhawk | -4.41 | -1.33 | 0 | 4.97E-04 |  | -4.41 | -1.29 | 0 | 4.46E-03 |
| Black Goshawk | -1.64 | 0 | 0 | 0 |  | -1.64 | 0 | 0 | 0 |
| Wahlberg's Eagle | -10.3 | 6.36 | 0 | 7.78E-04 |  | -10 | 5.26E-07 | -9.6E-15 | 2.76 |
|  |  |  |  |  |  |  |  |  |  |
| Table S3 cont. |  |  |  |  |  |  |  |  |  |
| Common Name | Food Energy (GJ ha^-1^ year^-1^) | | | |  | Income (US$ ha^-1^ year^-1^) | | | |
|  | b0 | b1 | b2 | alpha |  | b0 | b1 | b2 | alpha |
| African Hawk-eagle | -113 | 6.34 | -0.0904 | 1.77 |  | -158 | 0.000739 | -8.8E-10 | 2.15 |
| Long-crested Eagle | -3.75 | -0.00052 | 0 | 2.84 |  | -3.72 | -0.00105 | 0 | 0.973 |
| Crowned Hawk-eagle | -3.72 | 0 | 0 | 0 |  | -3.72 | 0 | 0 | 0 |
| Black-headed Heron | -11.1 | 7.61 | 0 | 3.22E-02 |  | -10.4 | 6.99 | 0 | 1.14E-02 |
| Cattle Egret | -5.93 | 1.21 | 0 | 0.502 |  | -3.98 | 4.23E-08 | 0 | 2.83 |
| Hamerkop | -10.5 | 5.86 | 0 | 5.72E-04 |  | -12.6 | 6.84 | 0 | 2.56E-02 |
| Hadada Ibis | -6.96 | 1.66 | -0.127 | 0.741 |  | -21 | 35.5 | -15 | 7.19E-02 |
| Marabou Stork | -8.29 | 0.288 | -0.00379 | 1.43 |  | -11.1 | 0.573 | 0 | 0.406 |
| Grey-backed Fiscal | -6.88 | 0.000378 | -5.7E-09 | 4.19 |  | -21.1 | 1.36E-06 | -2.3E-14 | 2.78 |
| Common Fiscal | -94.3 | 9.5 | -0.246 | 1.86 |  | -218 | 0.000951 | -1.1E-09 | 2.1 |
| Western Black-headed Oriole | -1.45 | 0 | 0 | 0 |  | -1.45 | 0 | 0 | 0 |
| African Black-headed Oriole | -25.9 | 26.8 | -7.58 | 0.266 |  | -13.2 | 0.121 | -0.00033 | 0.87 |
| Fork-tailed Drongo | -8.45 | 2.09 | -0.157 | 0.862 |  | -6.6 | 5.63E-07 | -1.5E-14 | 2.72 |
| Velvet-mantled Drongo | -1.74 | 0 | 0 | 0 |  | -1.74 | 0 | 0 | 0 |
| Pied Crow | -5.47 | 0.000646 | -3.6E-08 | 3.87 |  | -14.7 | 9.98E-07 | -2.1E-14 | 2.72 |
| Black Cuckooshrike | -2.19 | 0 | 0 | 0 |  | -2.19 | 0 | 0 | 0 |
| Red-shouldered Cuckooshrike | -10.2 | 5.44 | -0.883 | 1.52 |  | -221 | 0.000881 | -8.8E-10 | 2.24 |
| Purple-throated Cuckooshrike | -2.75 | 0 | 0 | 0 |  | -2.75 | 0 | 0 | 0 |
| African Blue-flycatcher | -1.27 | 1.42 | 0 | 2.86E-04 |  | -1.27 | 1.39 | 0 | 4.40E-03 |
| Dusky Crested-flycatcher | -2.28 | 0 | 0 | 0 |  | -2.28 | 0 | 0 | 0 |
| Blue-headed Crested-flycatcher | -3.99 | 0 | 0 | 0 |  | -3.99 | 0 | 0 | 0 |
| African Paradise-flycatcher | -8.42 | 7.26 | 0 | 5.57E-03 |  | -7.89 | 6.73 | 0 | 2.00E-03 |
| Black-headed Paradise-flycatcher | -1.72 | 0 | 0 | 0 |  | -1.72 | 0 | 0 | 0 |
|  |  |  |  |  |  |  |  |  |  |
| Table S3 cont. |  |  |  |  |  |  |  |  |  |
| Common Name | Food Energy (GJ ha^-1^ year^-1^) | | | |  | Income (US$ ha^-1^ year^-1^) | | | |
|  | b0 | b1 | b2 | alpha |  | b0 | b1 | b2 | alpha |
| Northern Puffback | -2.89 | 0.00427 | -2.2E-06 | 2.38 |  | -3.19 | 0.0113 | 0 | 0.753 |
| Black-crowned Tchagra | -26.5 | 10.6 | -1.19 | 0.703 |  | -12.3 | 8.4 | 0 | 1.80E-04 |
| Brown-crowned Tchagra | -27 | 25.7 | -6.42 | 0.354 |  | -8.49 | 6.53 | 0 | 1.43E-03 |
| Ethiopian Boubou | -9.63 | 25.6 | -17.5 | 4.56E-02 |  | -45.5 | 4.59 | -0.121 | 0.486 |
| Black-headed Gonolek | -32.7 | 21.7 | -3.95 | 0.449 |  | -89.6 | 0.135 | -5.2E-05 | 1.18 |
| Sooty Boubou | -2.41 | 0 | 0 | 0 |  | -2.41 | 0 | 0 | 0 |
| Grey-green Bush-shrike | -4.3 | -0.00099 | 0 | 3.92 |  | -4.51 | -3.3E-10 | 0 | 3.56 |
| African Shrike-flycatcher | -1.91 | 0 | 0 | 0 |  | -1.91 | 0 | 0 | 0 |
| Black-and-white Shrike-flycatche | -1.92 | -3.3E-05 | 1.01E-10 | 3.98 |  | -1.66 | -1.7E-12 | 0 | 4.33 |
| Brown-throated Wattle-eye | -1.38 | 0.432 | 0 | 8.67E-04 |  | -1.36 | 0.0556 | -0.00106 | 0.612 |
| Chestnut Wattle-eye | -2.69 | 0 | 0 | 0 |  | -2.69 | 0 | 0 | 0 |
| Jameson's Wattle-eye | -2.38 | 0 | 0 | 0 |  | -2.38 | 0 | 0 | 0 |
| Rufous Flycatcher-thrush | -2.99 | 0 | 0 | 0 |  | -2.99 | 0 | 0 | 0 |
| African Thrush | -2.66 | 1.39 | -0.157 | 0.624 |  | -2.51 | 0.00103 | -9.4E-08 | 1.4 |
| Brown-chested Alethe | -2.17 | 0 | 0 | 0 |  | -2.17 | 0 | 0 | 0 |
| White-tailed Alethe | -3.35 | 0 | 0 | 0 |  | -3.35 | 0 | 0 | 0 |
| Northern Black Flycatcher | -3.69 | 2.09 | -0.42 | 0.482 |  | -3.64 | 1.17 | -0.115 | 0.328 |
| African Dusky Flycatcher | -3.54 | 0.00475 | -3.1E-06 | 2.97 |  | -4.64 | 3.76E-07 | -1.2E-14 | 2.7 |
| Dusky-blue Flycatcher | -4.51 | 0 | 0 | 0 |  | -4.51 | 0 | 0 | 0 |
| Ashy Flycatcher | -1.48 | -2.6 | 0 | 0.18 |  | -1.48 | -0.0567 | 0 | 0.687 |
| Grey-throated Tit-flycatcher | -1.75 | 0 | 0 | 0 |  | -1.75 | 0 | 0 | 0 |
| Grey Tit-flycatcher | -2.49 | 0 | 0 | 0 |  | -2.49 | 0 | 0 | 0 |
| Blue-shouldered Robin-chat | -1.49 | 0 | 0 | 0 |  | -1.49 | 0 | 0 | 0 |
|  |  |  |  |  |  |  |  |  |  |
| Table S3 cont. |  |  |  |  |  |  |  |  |  |
| Common Name | Food Energy (GJ ha^-1^ year^-1^) | | | |  | Income (US$ ha^-1^ year^-1^) | | | |
|  | b0 | b1 | b2 | alpha |  | b0 | b1 | b2 | alpha |
| White-browed Robin-chat | -590 | 1060 | -481 | 4.99E-02 |  | -50.7 | 3.47 | -0.0612 | 0.548 |
| Red-capped Robin-chat | -2.77 | 0 | 0 | 0 |  | -2.77 | 0 | 0 | 0 |
| Snowy-crowned Robin-chat | -2.39 | -2.18 | 0 | 2.02E-02 |  | -2.39 | -2.27 | 0 | 1.71E-04 |
| Brown-backed Scrub Robin | -12 | 8.82 | 0 | 4.32E-02 |  | -10.5 | 6.95 | 0 | 2.83E-02 |
| Red-backed Scrub-robin | -7.57 | 1.15 | -0.0652 | 1.17 |  | -164 | 0.00104 | -1.7E-09 | 2.11 |
| Forest Robin | -1.24 | 0 | 0 | 0 |  | -1.24 | 0 | 0 | 0 |
| Whinchat | -6.51 | 0.00656 | -3.4E-06 | 3.06 |  | -10.7 | 6.11 | 0 | 1.78E-03 |
| Sooty Chat | -21.1 | 14.4 | -2.67 | 0.439 |  | -7.72 | 5.68E-07 | -1.2E-14 | 2.74 |
| Chestnut-winged Starling | -4.78 | 0 | 0 | 0 |  | -4.78 | 0 | 0 | 0 |
| Purple-headed Glossy-starling | -1.43 | 0 | 0 | 0 |  | -1.43 | 0 | 0 | 0 |
| Splendid Glossy-starling | -0.166 | 13.4 | -11.9 | 9.65E-02 |  | -0.154 | -1.5E-10 | 0 | 3.68 |
| Rueppell's Glossy-starling | -10.3 | 6.51 | -1.17 | 0.447 |  | -17.9 | 0.823 | -0.0102 | 0.616 |
| Violet-backed Starling | -1 | -1.28 | 0 | 0.316 |  | -0.949 | -3E-12 | 0 | 4.6 |
| African Penduline-tit | -3.64 | 0 | 0 | 0 |  | -3.64 | 0 | 0 | 0 |
| White-winged Tit | -10.9 | 8.87 | 0 | 1.13E-04 |  | -10.8 | 8.73 | 0 | 2.91E-04 |
| White-winged Tit | -3.29 | 0 | 0 | 0 |  | -3.29 | 0 | 0 | 0 |
| Dusky Tit | -2.89 | 0 | 0 | 0 |  | -2.89 | 0 | 0 | 0 |
| Common Bulbul | 0.612 | 1.44 | -0.261 | 0.366 |  | 0.619 | 0.548 | 0 | 0.198 |
| Little Greenbul | 2.32 | -0.998 | 0 | 0.445 |  | 2.32 | -0.00092 | 0 | 1.29 |
| Grey Greenbul | -2.76 | 0 | 0 | 0 |  | -2.76 | 0 | 0 | 0 |
| Plain Greenbul | -1.21 | 0 | 0 | 0 |  | -1.21 | 0 | 0 | 0 |
| Slender-billed Greenbul | -2.49 | 0 | 0 | 0 |  | -2.49 | 0 | 0 | 0 |
| Yellow-whiskered Greenbul | -2.01 | 0 | 0 | 0 |  | -2.01 | 0 | 0 | 0 |
|  |  |  |  |  |  |  |  |  |  |
| Table S3 cont. |  |  |  |  |  |  |  |  |  |
| Common Name | Food Energy (GJ ha^-1^ year^-1^) | | | |  | Income (US$ ha^-1^ year^-1^) | | | |
|  | b0 | b1 | b2 | alpha |  | b0 | b1 | b2 | Alpha |
| Honeyguide Greenbul | -2.94 | 0 | 0 | 0 |  | -2.94 | 0 | 0 | 0 |
| Yellow-throated Greenbul | -2.32 | -0.42 | 0 | 0.992 |  | -2.19 | -0.72 | 0 | 0.23 |
| Joyful Greenbul | -3.09 | 0 | 0 | 0 |  | -3.09 | 0 | 0 | 0 |
| Toro Olive Greenbul | -1.94 | 0 | 0 | 0 |  | -1.94 | 0 | 0 | 0 |
| White-throated Greenbul | -1.73 | 0 | 0 | 0 |  | -1.73 | 0 | 0 | 0 |
| Common Bristlebill | -3.3 | 0 | 0 | 0 |  | -3.3 | 0 | 0 | 0 |
| Lesser Bristlebill | -3.4 | 0 | 0 | 0 |  | -3.4 | 0 | 0 | 0 |
| Yellow-spotted Nicator | -2.94 | 0 | 0 | 0 |  | -2.94 | 0 | 0 | 0 |
| Red-tailed Bulbul | -2.15 | 0 | 0 | 0 |  | -2.15 | 0 | 0 | 0 |
| Red-faced Cisticola | -80 | 149 | -69.7 | 5.01E-02 |  | -7.87 | 7.17 | 0 | 8.06E-03 |
| Winding Cisticola | -8.78 | 0.000149 | -5.9E-10 | 4.56 |  | -14.9 | 9.96E-07 | -1.8E-14 | 2.72 |
| Croaking Cisticola | -25 | 26 | -7.15 | 0.259 |  | -11.4 | 8.36 | 0 | 2.42E-02 |
| Tawny-flanked Prinia | -6.2 | 7.18 | 0 | 1.76E-02 |  | -6.96 | 6.97 | 0 | 2.66E-02 |
| White-chinned Prinia | -3.01 | -1.4 | 0 | 1.65E-04 |  | -2.96 | -2E-10 | 0 | 3.74 |
| Black-throated Apalis | -1.62 | 0 | 0 | 0 |  | -1.62 | 0 | 0 | 0 |
| Buff-throated Apalis | -1.12 | 0 | 0 | 0 |  | -1.12 | 0 | 0 | 0 |
| Grey-capped Warbler | -94.1 | 7.57 | -0.157 | 1.77 |  | -188 | 133 | 0 | 5.01E-02 |
| Green-backed Camaroptera | 0.565 | 1.03 | -0.211 | 0.433 |  | 0.559 | 1.02 | 0 | 2.19E-02 |
| Yellow-browed Camaroptera | -1.01 | 0 | 0 | 0 |  | -1.01 | 0 | 0 | 0 |
| Olive-green Camaroptera | -2.78 | 0 | 0 | 0 |  | -2.78 | 0 | 0 | 0 |
| African Yellow White-eye | 0.59 | 0.371 | 0 | 0.294 |  | 0.585 | 0.000164 | 0 | 1.35 |
| Black-faced Rufous Warbler | -4.68 | 0 | 0 | 0 |  | -4.68 | 0 | 0 | 0 |
| Moustached Grass-warbler | -41.4 | 23.7 | -3.59 | 0.579 |  | -62 | 2.63 | -0.0292 | 0.621 |
|  |  |  |  |  |  |  |  |  |  |
| Table S3 cont. |  |  |  |  |  |  |  |  |  |
| Common Name | Food Energy (GJ ha^-1^ year^-1^) | | | |  | Income (US$ ha^-1^ year^-1^) | | | |
|  | b0 | b1 | b2 | alpha |  | b0 | b1 | b2 | alpha |
| Eastern Olivaceous Warbler | -14.5 | 9.45 | 0 | 1.69E-02 |  | -183 | 7.99 | -0.0891 | 0.626 |
| Icterine Warbler | -38.4 | 23.3 | -3.87 | 0.553 |  | -13 | 8.56 | 0 | 3.77E-05 |
| Buff-bellied Warbler | -25.1 | 10.5 | -1.18 | 1.78 |  | -168 | 0.000673 | -6.9E-10 | 2.24 |
| Green Crombec | -1.9 | 0 | 0 | 0 |  | -1.9 | 0 | 0 | 0 |
| Northern Crombec | -4.68 | 0 | 0 | 0 |  | -4.68 | 0 | 0 | 0 |
| Red-faced Crombec | -11 | 2.74 | 0 | 0.315 |  | -21.1 | 0.221 | 0 | 0.678 |
| Yellow Longbill | -2.19 | 0 | 0 | 0 |  | -2.19 | 0 | 0 | 0 |
| Grey Longbill | -2.27 | 0 | 0 | 0 |  | -2.27 | 0 | 0 | 0 |
| Green Hylia | -2.32 | 0 | 0 | 0 |  | -2.32 | 0 | 0 | 0 |
| Willow Warbler | -3.51 | -0.00024 | 0 | 3.63 |  | -3.3 | -6.5E-13 | 0 | 4.6 |
| Wood Warbler | -3.29 | 0 | 0 | 0 |  | -3.29 | 0 | 0 | 0 |
| Scaly-breasted Illadopsis | -1.8 | 0 | 0 | 0 |  | -1.8 | 0 | 0 | 0 |
| Pale-breasted Illadopsis | -1.27 | 0 | 0 | 0 |  | -1.27 | 0 | 0 | 0 |
| Brown Illadopsis | -2.31 | 0 | 0 | 0 |  | -2.31 | 0 | 0 | 0 |
| Sharpe's Pied-babbler | -23.1 | 8.34 | -0.811 | 0.865 |  | -42.1 | 0.162 | -0.00016 | 1.01 |
| Brown Babbler | -183 | 9.63 | -0.128 | 1.8 |  | -160 | 0.000716 | -8.2E-10 | 2.15 |
| Arrow-marked Babbler | -275 | 6 | -0.0328 | 1.81 |  | -176 | 0.000673 | -6.5E-10 | 2.14 |
| Garden Warbler | -510 | 7.88 | -0.0305 | 1.83 |  | -215 | 0.00104 | -1.3E-09 | 2.01 |
| Flappet Lark | -6 | 1.33E-06 | 0 | 4.6 |  | -6.77 | 2.08E-10 | 0 | 3.62 |
| Scarlet-tufted Sunbird | -1.48 | 0 | 0 | 0 |  | -1.48 | 0 | 0 | 0 |
| Green Sunbird | -1.14 | 0 | 0 | 0 |  | -1.14 | 0 | 0 | 0 |
| Collared Sunbird | -2 | 0 | 0 | 0 |  | -2 | 0 | 0 | 0 |
| Little Green Sunbird | -1.65 | 0 | 0 | 0 |  | -1.65 | 0 | 0 | 0 |
|  |  |  |  |  |  |  |  |  |  |
| Table S3 cont. |  |  |  |  |  |  |  |  |  |
| Common Name | Food Energy (GJ ha^-1^ year^-1^) | | | |  | Income (US$ ha^-1^ year^-1^) | | | |
|  | b0 | b1 | b2 | alpha |  | b0 | b1 | b2 | alpha |
| Olive Sunbird | -1.29 | 0 | 0 | 0 |  | -1.29 | 0 | 0 | 0 |
| Green-headed Sunbird | -0.592 | -0.276 | 0 | 0.483 |  | -0.561 | -0.00171 | 0 | 1.01 |
| Blue-throated Brown Sunbird | -0.395 | 0 | 0 | 0 |  | -0.395 | 0 | 0 | 0 |
| Green-throated Sunbird | -1.15 | 0 | 0 | 0 |  | -1.15 | 0 | 0 | 0 |
| Scarlet-chested Sunbird | -1.77 | 1.78 | -0.295 | 0.467 |  | -1.85 | 2.56 | 0 | 1.84E-03 |
| Variable Sunbird | -75.9 | 142 | -65.5 | 5.01E-02 |  | -23.9 | 27.5 | -7.59 | 0.116 |
| Northern Double-collared Sunbird | -631 | 1130 | -509 | 5.04E-02 |  | -18.4 | 0.0885 | -0.00011 | 0.988 |
| Olive-bellied Sunbird | -1.7 | 1.33 | 0 | 4.55E-03 |  | -1.7 | 1.34 | 0 | 6.10E-04 |
| Copper Sunbird | -9.01 | 7.42 | 0 | 1.88E-02 |  | -11.2 | 6.5 | 0 | 6.78E-02 |
| Bronze Sunbird | -251 | 10.2 | -0.104 | 1.8 |  | -93.4 | 64.6 | 0 | 4.96E-02 |
| Red-chested Sunbird | -7.02 | 22.9 | -15.7 | 2.75E-02 |  | -13.5 | 9.71 | 0 | 5.00E-02 |
| Mariqua Sunbird | -12.6 | 9.91 | 0 | 3.34E-02 |  | -11.2 | 9.21 | 0 | 4.29E-04 |
| Superb Sunbird | -1.84 | 0 | 0 | 0 |  | -1.84 | 0 | 0 | 0 |
| Northern Grey-headed Sparrow | -6.57 | 7.43 | 0 | 2.08E-02 |  | -7.21 | 6.84 | 0 | 3.33E-02 |
| African Pied Wagtail | -29 | 22.2 | -4.52 | 0.448 |  | -9.23 | 6.6 | 0 | 2.33E-03 |
| Yellow Wagtail | -11.7 | 6.01 | 0 | 7.30E-02 |  | -11.1 | 6.37 | 0 | 3.11E-03 |
| Yellow-throated Longclaw | -9.16 | 6.33 | 0 | 4.12E-04 |  | -9.04 | 6.15 | 0 | 9.40E-03 |
| Baglafecht Weaver | -8.29 | 6.68 | 0 | 1.08E-03 |  | -9.26 | 2.74 | 0 | 0.166 |
| Spectacled Weaver | -6.04 | 1.71E-06 | 0 | 4.6 |  | -7.6 | 5.92E-09 | 0 | 3.17 |
| Black-necked Weaver | -0.337 | -1.91 | 0 | 0.292 |  | -0.338 | -0.00507 | 0 | 1.07 |
| Vieillot's Black Weaver | -1.71 | 0.00454 | -4.5E-06 | 2.83 |  | -1.69 | 1.77E-08 | -8.8E-17 | 3.03 |
| Weyns's Weaver | 0.771 | 0 | 0 | 0 |  | 0.771 | 0 | 0 | 0 |
| Black-headed Weaver | -84.9 | 151 | -66.1 | 4.98E-02 |  | -5.51 | 0.121 | -0.00053 | 0.769 |
|  |  |  |  |  |  |  |  |  |  |
| Table S3 cont. |  |  |  |  |  |  |  |  |  |
| Common Name | Food Energy (GJ ha^-1^ year^-1^) | | | |  | Income (US$ ha^-1^ year^-1^) | | | |
|  | b0 | b1 | b2 | alpha |  | b0 | b1 | b2 | alpha |
| Golden-backed Weaver | -103 | 8.27 | -0.167 | 1.79 |  | -212 | 0.00106 | -1.3E-09 | 2.01 |
| Yellow-mantled Weaver | -1.45 | 0 | 0 | 0 |  | -1.45 | 0 | 0 | 0 |
| Red-headed Malimbe | -2.19 | -0.0977 | 0 | 3 |  | -2.19 | -2.5E-08 | 0 | 3.17 |
| Black Bishop | -81.3 | 12.1 | -0.452 | 1.79 |  | -172 | 0.000716 | -7.5E-10 | 2.14 |
| Black-winged Bishop | -89 | 0.0155 | 0 | 2.71 |  | -196 | 6.83 | 0 | 0.514 |
| Fan-tailed Widowbird | -2.9 | -3E-06 | 1.84E-12 | 4.6 |  | -3.38 | -2.1E-09 | 7.14E-17 | 2.95 |
| Yellow-shouldered Widowbird | -196 | 0.0334 | -1.4E-06 | 4.35 |  | -94.1 | 65.4 | 0 | 4.92E-02 |
| Grosbeak Weaver | -1.3 | -2.81 | 0 | 1.03E-05 |  | -1.3 | -2.81 | 0 | 3.25E-05 |
| White-breasted Negrofinch | -1.35 | 0 | 0 | 0 |  | -1.35 | 0 | 0 | 0 |
| Grey-headed Negrofinch | -1.39 | -1.03 | 0 | 0.571 |  | -1.39 | -0.00176 | 0 | 1.22 |
| Green-winged Pytilia | -31.6 | 22.7 | -4.39 | 0.45 |  | -10.3 | 1.15E-06 | -3.9E-14 | 2.72 |
| Green-backed Twinspot | -2 | 0 | 0 | 0 |  | -2 | 0 | 0 | 0 |
| Black-bellied Seedcracker | -1.68 | 0 | 0 | 0 |  | -1.68 | 0 | 0 | 0 |
| Red-headed Bluebill | -1.3 | 0 | 0 | 0 |  | -1.3 | 0 | 0 | 0 |
| Red-billed Firefinch | -300 | 546 | -248 | 5.00E-02 |  | -21.6 | 0.782 | -0.00681 | 0.659 |
| African Firefinch | -13 | 9.64 | 0 | 2.21E-02 |  | -12.2 | 8.53 | 0 | 1.43E-02 |
| Red-cheeked Cordonbleu | -6.23 | 3.06 | -0.328 | 0.499 |  | -47.3 | 34.8 | 0 | 4.99E-02 |
| Crimson-rumped Waxbill | -475 | 903 | -430 | 4.98E-02 |  | -62.8 | 2.83 | -0.0328 | 0.63 |
| Common Waxbill | -34.9 | 19.9 | -2.98 | 0.665 |  | -8.02 | 5.55E-07 | -1.3E-14 | 2.73 |
| Black-crowned Waxbill | -113 | 212 | -98 | 5.00E-02 |  | -9.18 | 7.79 | 0 | 4.74E-02 |
| Bronze Munia | -6.09 | 10.2 | -2.93 | 0.207 |  | -5.19 | 0.603 | -0.0115 | 0.531 |
| Black-and-white Munia | -21.9 | 24.7 | -6.96 | 0.299 |  | -3.62 | 1.13E-05 | -8.6E-12 | 2.17 |
| Magpie Munia | -3.5 | 0 | 0 | 0 |  | -3.5 | 0 | 0 | 0 |
|  |  |  |  |  |  |  |  |  |  |
| Table S3 cont. |  |  |  |  |  |  |  |  |  |
| Common Name | Food Energy (GJ ha^-1^ year^-1^) | | | |  | Income (US$ ha^-1^ year^-1^) | | | |
|  | b0 | b1 | b2 | alpha |  | b0 | b1 | b2 | alpha |
| Village Indigobird | -4.28 | 0.284 | -0.00591 | 1.33 |  | -6.26 | 7.41E-07 | -2.4E-14 | 2.69 |
| Pin-tailed Whydah | -8.57 | 5.6 | -1.1 | 0.499 |  | -27.1 | 0.576 | -0.00322 | 0.741 |
| Yellow-browed Citril | -11.5 | 8.57 | 0 | 2.34E-05 |  | -8.5 | 6.73 | -1.37 | 0.223 |
| Black-throated Seedeater | -3.9 | 0.000104 | -7.2E-10 | 4.6 |  | -7.21 | 4.04E-07 | -6.5E-15 | 2.78 |
| Yellow-fronted Canary | -234 | 424 | -190 | 5.01E-02 |  | -9.32 | 7.9 | 0 | 4.62E-02 |
| African Golden-breasted Bunting | -7.72 | 4.12 | -0.717 | 0.521 |  | -7.18 | 0.000227 | -2.3E-09 | 1.75 |
