# Supplementary material for: Conserving the Birds of Uganda’s Banana-Coffee Arc: Land Sparing and Land Sharing Compared
Source: PLoS One. 2013 Feb 4;8(2):e54597. doi: 10.1371/journal.pone.0054597 (PMC3563584; doi:10.1371/journal.pone.0054597)
Supplement: Text S1 — The effect of a settling period on the number of individuals seen in the ten minute point count period at forest sites. (DOCX) [file pone.0054597.s004.docx]

Hulme et al. Supplementary material Text S1

The effect of a settling period on the number of individuals seen in the ten minute point count period at forest sites was tested by comparing the total number of registrations per point on the prelinary surveys without a settling period (usually only one point per site) to the same points carried out on the first visit (i.e. closest in date) of the main survey, which included a two minute settling period. Differences (preliminary – main survey) were tested for significance using paired t-tests where a given species was recorded on a minimum of five sites (the site was the basic analytical unit – where more than one point was surveyed for the preliminary survey, data were summed and compared with the same points from the main surveys).

There were eight species (out of 61) that showed a significant difference. With one exception (Western Nicator) the differences were only weakly significant (P > 0.025). Applying Bonferroni corrections for multiple testing would give a significance level of P = 0.0008, thus all significant differences may have arisen by chance. This is further supported by the fact that the direction of the effect was not consistent – there were three species where counts were higher without the settling period, and five where it was lower. Of the eight species in Table S1 six were only recorded at forest sites so only two, Eastern-grey Plantain-eater and Black &White Casqued Hornbill, had the potential for differences in methods to affect their density-yield function at yields above zero. We therefore conclude that there was at most a weak effect of settling period for the forest surveys.
